# New Isoflavanes from *Spatholobus suberectus* and Their Cytotoxicity against Human Breast Cancer Cell Lines

**DOI:** 10.3390/molecules24183218

**Published:** 2019-09-04

**Authors:** Fu Peng, Huan Zhu, Chun-Wang Meng, Yan-Rui Ren, Ou Dai, Liang Xiong

**Affiliations:** 1 West China School of Pharmacy, Sichuan University, Chengdu 610041, China; 2 School of Pharmacy, Chengdu University of Traditional Chinese Medicine, Chengdu 611137, China (H.Z.) (C.-W.M.) (Y.-R.R.); 3 Institute of Innovative Medicine Ingredients of Southwest Specialty Medicinal Materials, Chengdu University of Traditional Chinese Medicine, Chengdu 611137, China

**Keywords:** *Spatholobus suberectus*, isoflavanes, breast cancer, MCF-7, MDA-MB-231

## Abstract

The rattans of *Spatholobus suberectus* Dunn are a traditional Chinese medicine activating blood circulation and removing stasis. They have often been used for the traditional Chinese medicinal treatment of breast cancer in modern China. In this study, four novel isoflavanes (**1**–**3** and **5**) and four known analogues (**4** and **6**–**8**) were isolated from an ethanolic extract of the rattans of *S. suberectus*. Their structures were elucidated by extensive spectroscopic analyses and electronic circular dichroism studies. MCF-7 and MDA-MB-231 human breast cancer cell lines were used to evaluate the cytotoxic effects of the isolates. Interestingly, compounds **1** and **2** only inhibited the proliferation of MCF-7 cells, while compound **6** showed a selective cytotoxicity against MDA-MB-231 cells. However, compound **4** had significant cytotoxicity against both MCF-7 and MDA-MB-231 cell lines.

## 1. Introduction

*Spatholobus suberectus* Dunn (Leguminosae) is a representative traditional Chinese medicine historically used to promote blood circulation and remove stasis. As traditional Chinese medicine theory considers cancer as a kind of disease most possibly related to severe blood stasis, *S. suberectus* is commonly used for the treatment of cancer in China [1]. Modern studies on *S. suberectus* have indicated that its extracts and compounds have cytotoxic effects against human cancer cell lines, especially breast cancer cell lines [1,2,3]. Phytochemical investigations have found that *S. suberectus* contains flavonoids, sterols, lignans, anthraquinones, phenolic acids, terpenoids, and their glycosides [4,5,6,7,8]. Flavonoids are reported to be the main active secondary metabolites, including flavones, flavanones, isoflavanes, isoflavones, and chalcones [2,4,9,10]. Some of them, such as genistein and gallocatechin, have been demonstrated to be effective for cancer prevention or treatment [11]. In our previous study, a series of chalcones and flavanones were isolated from *S. suberectus* and synthesized. The cytotoxicity against two breast cancer cell lines (MCF-7 and MDA-MB-231) and the structure–cytotoxicity relationship were studied. A methoxylated chalcone, 3′,4′,5′,4″-tetramethoxychalcone, was found to be most effective [9]. To further explore the cytotoxic components of *S. suberectus* against breast cancer cells, the same ethanolic extract of the rattans of *S. suberectus* was continuously investigated.

**Figure 1:**
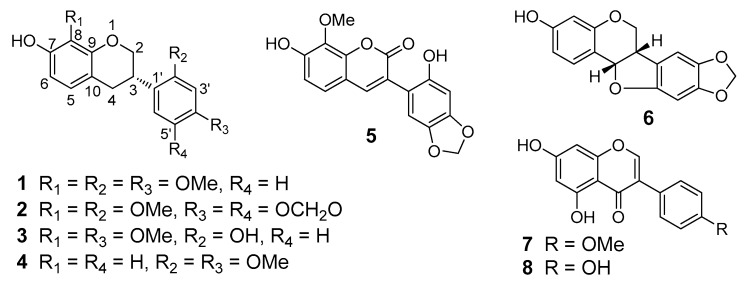
Chemical structures of compounds **1**–**8**.

Isoflavanes and isoflavones are two subclasses of isoflavonoids, which are plant secondary metabolites characterized by a B-ring attached at the C-3 position of the C-ring. They are known for their significant estrogen-like activity, and modern research suggests that they also have antiproliferation in many kinds of human cancer cell lines [12,13,14,15,16,17]. However, there are few reports on the cytotoxic isoflavanes and isoflavones of *S. suberectus*. In this study, eight isoflavonoid derivatives (**1**–**8**) (Figure 1), including four new isoflavanes (**1**–**3** and **5**), were isolated and characterized from *S. suberectus*. The cytotoxic effects of the isolates were determined by an MTT [3-(4,5-dimethylthiazol-2-yl)-2,5-diphenyltetrazolium bromide] colorimetric assay using an estrogen-dependent breast cancer cell line (MCF-7) and an estrogen-independent breast cancer cell line (MDA-MB-231). It is interesting that isoflavanes **1**, **2**, and **6** showed selective cytotoxicity, and isoflavane **4** inhibited the proliferation of both MCF-7 and MDA-MB-231 cell lines.

## 2. Results and Discussion

**Figure 2:**
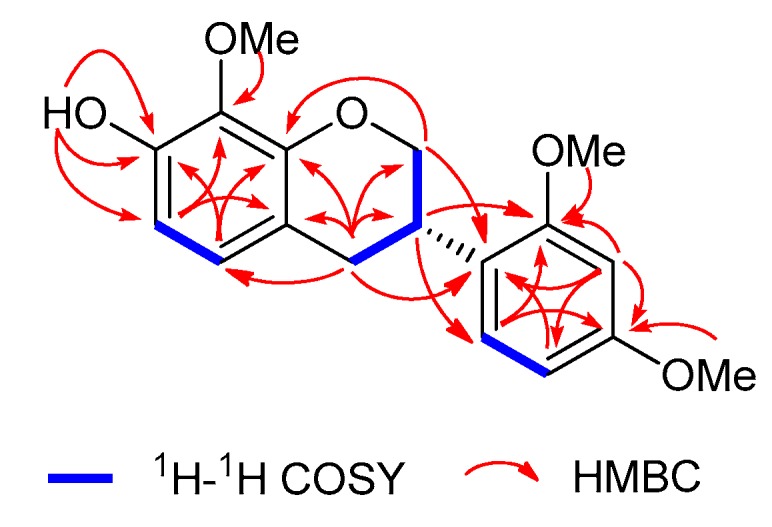
Key ^1^H–^1^H COSY and HMBC correlations of **1**.

Compound **1** was isolated as a white amorphous powder, and the presence of hydroxy groups (3446 cm^−1^) and aromatic rings (1614 and 1495 cm^−1^) was indicated in its infrared (IR) spectrum. High-resolution electrospray ionization mass spectrometry (HRESIMS) data at *m*/*z* 317.1389 [M + H]^+^ (calcd. for C_18_H_21_O_5_, 317.1389) and 339.1208 [M + Na]^+^ (calcd. for C_18_H_20_O_5_Na, 339.1208) revealed the molecular formula of C_18_H_20_O_5_ for **1**. The ^1^H nuclear magnetic resonance (NMR) spectrum of **1** displayed resonances corresponding to a 1,2,3,4-tetrasubstituted phenyl [*δ*_H_ 6.71 (d, *J* = 8.4 Hz) and 6.51 (d, *J* = 8.4 Hz)], a 1,2,4-trisubstituted phenyl [*δ*_H_ 7.02 (d, *J* = 8.4 Hz), 6.46 (dd, *J* = 8.4, 2.4 Hz), and 6.49 (d, *J* = 2.4 Hz)], two methylenes [*δ*_H_ 4.40 (ddd, *J* = 10.2, 3.6, 1.8 Hz), 4.03 (t, *J* = 10.2 Hz), 2.98 (ddd, *J* = 15.6, 11.4, 0.6 Hz), and 2.86 (ddd, *J* = 15.6, 4.8, 1.2 Hz)], a methine [*δ*_H_ 3.56 (m)], three methoxy groups (*δ*_H_ 3.91, 3.82, and 3.80), and an exchangeable hydroxy group (*δ*_H_ 5.63) (Table 1). The ^13^C-NMR and distortionless enhancement by polarization transfer (DEPT) spectra of **1** showed 18 carbon signals (Table 2) attributed to 3 × OCH_3_, 2 × CH_2_ (one oxygen-bearing), 6 × CH (five aromatic methines), and 7 × C (five oxygenated aromatic carbons). These spectroscopic data indicated that compound **1** was an isoflavane possessing a hydroxy and three methoxy groups [18,19,20]. Comparison of the ^1^H and ^13^C-NMR data of **1** and sativan [18] suggested that an additional methoxy group was substituted at C-8 in **1**. The planar structure of **1** was further confirmed by 2D NMR experiments, including ^1^H-detected heteronuclear single quantum coherence (HSQC), ^1^H–^1^H correlation spectroscopy (^1^H–^1^H COSY), and ^1^H-detected heteronuclear multiple bond (HMBC) experiments. Particularly, the HMBC correlations of H-5 with C-4, C-7, and C-9; of H-6 with C-7, C-8, and C-10; of OH-7 with C-6, C-7, and C-8; and of OMe-8 with C-8 (Figure 2), together with the ^1^H–^1^H COSY correlations of H-5/H-6, indicated that a hydroxy group and a methoxy group were substituted at C-7 and C-8, respectively. The locations of the other two methoxy groups (OMe-2′ and OMe-4′) were determined by the HMBC correlations of H-3′ with C-1′, C-2′, C-4′, and C-5′; of H-5′ with C-1′, C-3′, and C-4′; of H-6′ with C-3, C-2′, and C-4′; of OMe-2′ with C-2′; and of OMe-4′ with C-4′. The absolute configuration of **1** was established by electronic circular dichroism (ECD) data. A positive Cotton effect at 238 nm and a negative Cotton effect at 284 nm indicated the 3*S*-configuration for **1** [21]. Therefore, compound **1** was determined to be (3*S*)-7-hydroxy-8,2′,4′-trimethoxyisoflavane.

Compound **2** had similar spectroscopic data to **1**. The molecular formula of C_18_H_18_O_6_ determined by HRESIMS indicated that **2** possessed one more oxygen atom and two less hydrogen atoms than **1**. Comparison of the ^1^H and ^13^C-NMR data of **2** and **1** (Table 1 and Table 2) suggested that a 1,2,4,5-tetrasubstituted phenyl [*δ*_H_ 6.58 (s) and 6.66 (s); *δ*_C_ 95.0 (CH) and 107.2 (CH)] and a methylenedioxy [*δ*_H_ 6.90 (2H, s); *δ*_C_ 101.3 (CH_2_)] in **2** replaced the 1,2,4-trisubstituted phenyl and the OMe-4′ in **1**, respectively. In the HMBC spectrum of **2**, correlations of H-3′ with C-1′, C-2′, C-4′, and C-5′; of H-6′ with C-3, C-2′, C-4′, and C-5′; of OMe-2′ with C-2′; and of 4′-OCH_2_O-5′ with C-4′ and C-5′ confirmed that the methylenedioxy group in **2** was linked to C-4′ and C-5′. The ECD spectrum of **2** exhibited similar Cotton effects [CD (MeOH) 242 (Δ*ε* +1.86), 283 (Δ*ε* −0.62) nm] to **1**. Although the specific optical rotation ([α]${}_{D}^{25}$) of **2** was opposite to that of **1**, the ECD Cotton effects of **2** revealed that it also had a 3*S*-configuration [21]. Consequently, compound **2** was determined to be (3*S*)-7-hydroxy-8,2′-dimethoxy-4′,5′-methylenedioxyisoflavane.

Compound **3** was another isoflavane, as indicated by the HRESIMS, IR, ^1^H-NMR, and ^13^C-NMR data. Comparison of the ^1^H and ^13^C-NMR data of **3** and **1** suggested that the OMe-2′ in **1** was replaced by a hydroxy group in **3**. Thus, the planar structure of **3** was established as 7,2′-dihydroxy-8,4′-dimethoxyisoflavane (8-methoxyvestitol), which was reported in 1979 [22] and 2010 [23]. However, its absolute configuration was undescribed. In addition, the chemical shifts were very different between compound **3** and the reported 8-methoxyvestitol in reference [23], especially the chemical shifts of H-6′ (*δ*_H_ 6.34 in the reported 8-methoxyvestitol, 7.02 in compound **3**). No ^13^C-NMR or 2D-NMR data could be found for the reported 8-methoxyvestitol in reference [23]. To conclusively determine the structure of **3**, 2D-NMR (^1^H–^1^H COSY, HSQC, HMBC, and nuclear Overhauser enhancement spectroscopy (NOESY)) and ECD experiments were performed. Particularly, HMBC correlations of H-3′ with C-1′, C-2′, C-4′, and C-5′; of H-5′ with C-1′, C-3′, and C-4′; of H-6′ with C-3, C-2′, and C-4′; and of OMe-4′ with C-4′ confirmed the presence of ring B with OH-2′ and OMe-4′ groups. In the NOESY spectrum, correlations of OMe-4′ with H-3′ and H-5′ further verified the location of the OMe group. The 3*S*-configuration of **3** was substantiated also by ECD data (CD (MeOH) 234 (Δ*ε* +2.43), 284 (Δ*ε* −0.50) nm), as described above for **1** and **2**. Therefore, compound **3** was determined to be (3*S*)-7,2′-dihydroxy-8,4′-dimethoxyisoflavane.

Compound **5** was obtained as a yellow powder. Its molecular formula, C_17_H_12_O_7_, with 12 degrees of unsaturation, was deduced by HRESIMS. The ^1^H-NMR spectrum of **5** (Table 1) showed signals attributed to a 1,2,3,4-tetrasubstituted phenyl and a 1,2,4,5-tetrasubstituted phenyl, together with an aromatic methoxy, two aromatic hydroxys, and a methylenedioxy. Meanwhile, the ^1^H and ^13^C-NMR spectra showed characteristic signals for an α,β-unsaturated lactone [*δ*_H_ 7.79 (s); *δ*_C_ 144.2, 123.4, and 162.8] [24]. These spectroscopic data revealed that compound **5** was an isoflavane derivative of compound **2**. The main difference between **5** and **2** was that the ring C in **5** changed to a six-membered α,β-unsaturated lactone moiety. Additionally, a hydroxy group in **5** replaced the OMe-2′ in **2**. The above deduction was verified by 2D NMR data analysis. Particularly, the HMBC correlations from H-4 to C-2, C-5, C-9, and C-1′, together with their chemical shifts, confirmed the substructure of ring C. Therefore, compound **5** was determined to be 7,2′-dihydroxy-8-methoxy-4′,5′-methylenedioxyisoflavan-3-en-2-one.

Four known compounds were identified by comparison of the spectroscopic data obtained with those reported in the corresponding literature, as (*S*)-sativan (**4**) [18], maackiain (**6**) [25], biochanin A (**7**) [26], and genistein (**8**) [27].

As the extracts and the main compounds of *S. suberectus* have been reported to have cytotoxic activities against human breast cancer cells [1,2,3,9], all the isolates in this study were assayed for their cytotoxicity using an estrogen-dependent breast cancer cell line (MCF-7) and an estrogen-independent breast cancer cell line (MDA-MB-231). Four compounds (**1**, **2**, **4**, and **6**) showed cytotoxic effects, with IC_50_ values less than 100 μM (Table 3). Notably, compounds **1** and **2** selectively inhibited the proliferation of the MCF-7 cells, while compound **3** was inactive, and compound **4** exhibited an inhibitory effect on the proliferation of both the MCF-7 and MDA-MB-231 cells. When compared to compound **1**, replacement of the OMe-4′ by a 4′-OCH_2_O-5′ unit in ring B (**2**) decreased the cytotoxicity against the MCF-7 cell line; demethylation of OMe-2′ (**3**) resulted in a significant loss of such cytotoxicity; demethoxylation of OMe-8 (**4**) led to a cytotoxic isoflavane inhibiting the proliferation of both the MCF-7 and MDA-MB-231 cell lines. In addition, compound **6** exhibited a selective inhibitory effect on the proliferation of the MDA-MB-231 cells. Further comparison of their structures and IC_50_ values found that all isoflavanes possessing the OMe-2′ group (**1**, **2**, and **4**) had cytotoxicity against the MCF-7 cell line, and such cytotoxicity was lost when the OMe-2′ transformed to the OH-2′; all isoflavanes without the OMe-8 group (**4** and **6**) had cytotoxicity against the MDA-MB-231 cell line.

Thus far, isoflavanes have been reported to have a wide range of activities, but few studies have been conducted on their anti-breast cancer effects. (3*S*)-3′-Hydroxy-8-methoxyvestitol [14], possessing a similar structure to compounds **1**–**4**, showed better cytotoxicity against MCF-7 cells (IC_50_ = 17.87 ± 0.30 μM) than **1**–**4**. It can be speculated that the substituents on the aromatic rings in isoflavanes play an important role in inhibiting the proliferation of breast cancer cells. Although several natural isoflavones showed strong antiproliferative effects on breast cancer cells [15,16,17], the two isoflavones **7** and **8** isolated from *S. suberectus* were inactive in this study (IC_50_ > 100 μM). It is worth investigating whether other isoflavanes and isoflavones of *S. suberectus* have anti-breast cancer effects. In addition, the previous studies of *S. suberectus* on the cytotoxic effects and mechanisms against human breast cancer cells mainly focused on the total flavonoid extract [1,3]. Thus, further studies of the cytotoxic chemical components of *S. suberectus*, especially various types of flavonoids, are necessary.

## 3. Materials and Methods

### 3.1. General Procedures

Optical rotations were measured using an Anton Paar MCP 200 automatic polarimeter (Anton Paar GmbH, Graz, Austria). IR spectra were recorded using an Agilent Cary 600 FT-IR microscope instrument (Agilent Technologies Inc., Santa Clara, CA, USA). ECD spectra were recorded on an Applied Photophysics Chirascan and Chirascan-plus circular dichroism spectrometer (Applied Photophysics Ltd, Leatherhead, England). NMR spectra were obtained using a Bruker AVIIIHD-600 NMR spectrometer (Bruker Corporation, Billerica, MA, USA). Solvent peaks were used as references. HRESIMS spectra were measured using a Synapt G2 HDMS instrument (Waters Corporation Milford, Milford, MA, USA). Column chromatography was performed using silica gel (200−300 mesh; Yantai Institute of Chemical Technology, Yantai, China), Sephadex LH-20 (Amersham Pharmacia Biotech AB, Uppsala, Sweden), and polyamide (Jiangsu Changfeng Chemical Co. Ltd, Nanjing, China). Semipreparative high-performance liquid chromatography (HPLC) was performed on a 1220 Infinity LC instrument (Agilent Technologies Inc., Santa Clara, CA, USA) equipped with an Ultimate (250 × 10 mm) preparative column packed with C_18_ (5 μm). Thin-layer chromatography (TLC) was performed using glass plates precoated with silica gel GF_254_ plates (Qingdao Marine Chemical Inc., Qingdao, China).

### 3.2. Plant Material

The rattans of *Spatholobus suberectus* were purchased from Sichuan Neautus Traditional Chinese Medicine Co., Ltd. (Chengdu, China) in June 2013 and identified by Prof. Fei Long (Chengdu University of Traditional Chinese Medicine, China). A voucher specimen (SS-130625) was deposited at State Key Laboratory Breeding Base of Systematic Research, Development and Utilization of Chinese Medicine Resources, Chengdu University of Traditional Chinese Medicine.

### 3.3. Extraction and Isolation

The rattans of *S. suberectus* (15 kg) were extracted with 95% EtOH (3 × 90 L) under reflux for 3 × 3 h. The EtOH extract was evaporated under reduced pressure to yield a dark red residue (1.1 kg). The residue was suspended in H_2_O and partitioned with EtOAc to produce a dried EtOAc fraction (410 g). The EtOAc fraction (100 g) was subjected to column chromatography on silica gel (CH_2_Cl_2_/MeOH, 1:0–0:1) to yield 13 major fractions (F1−F13). Fraction F5 (28 g) was separated by polyamide column chromatography (10%–90% MeOH in H_2_O), affording seven subfractions (F5-1−F5-7).

Fraction F5-1 was further divided into F5-1-1−F5-1-13 by silica gel column chromatography using a gradient solvent system (petroleum ether/ethyl acetate, 1:0−0:1). Successive purification of F5-1-3 by Sephadex LH-20 (CH_2_Cl_2_−MeOH, 1:1), preparative TLC (CH_2_Cl_2_−MeOH, 20:1), and reversed-phase semipreparative HPLC (80% MeOH in H_2_O) afforded **1** (7 mg) and **2** (2 mg).

Fraction F5-2 was separated into six fractions (F5-2-1−F5-2-6) by Sephadex LH-20 with CH_2_Cl_2_/MeOH (1:1) as the eluent. Fraction F5-2-2 was further fractionated by Sephadex LH-20 (70% MeOH in H_2_O) to yield nine subfractions (F5-2-2-1−F5-2-2-9). Compounds **3** (5 mg) and **5** (2 mg) were obtained from F5-2-2-4 by successive separation on preparative TLC (CH_2_Cl_2_/MeOH, 5:1) and reversed-phase semipreparative HPLC (60% MeOH in H_2_O). Subfraction F5-2-2-8 was successively separated by a silica gel column eluted by petroleum ether/EtOAc (2:1), preparative TLC developed by CH_2_Cl_2_/MeOH (5:1), and reversed-phase semipreparative HPLC (70% MeOH in H_2_O) to give **6** (11 mg). F5-2-3 was fractionated by column chromatography over silica gel (petroleum ether/Me_2_CO, 50:1–1:1), followed by preparative TLC (CH_2_Cl_2_/EtOAc, 12:1) and reversed-phase semipreparative HPLC (55% MeOH in H_2_O), to yield **4** (5 mg), **7** (11 mg), and **8** (29 mg).

*(3S)-7-Hydroxy-8,2′,4′-trimethoxyisoflavane* (**1**): White amorphous powder, [α]${}_{D}^{25}$ = −27.4 (*c* 0.08, MeOH); CD (MeOH) 238 (Δ*ε* +2.05), 284 (Δ*ε* −0.98) nm; IR *ν*_max_: 3446, 2939, 2839, 1614, 1495, 1462, 1287, 1201, 1173, 1151, 1088, 1036, 966, 928, 824, 803 cm^−1^; ^1^H-NMR data, see Table 1; ^13^C-NMR data, see Table 2; HRESI-MS: *m*/*z* 317.1389 [M + H]^+^ (calcd. for C_18_H_21_O_5_, 317.1389), 339.1208 [M + Na]^+^ (calcd. for C_18_H_20_O_5_Na, 339.1208), 355.0949 [M + K]^+^ (calcd. for C_18_H_20_O_5_K, 355.0948). The original HRESIMS, IR, and 1D- and 2D-NMR spectra of **1** were shown in Supplementary Materials.

*(3S)-7-Hydroxy-8,2′-dimethoxy-4′,5′-methylenedioxyisoflavane* (**2**): White amorphous powder, [α]${}_{D}^{25}$ = +11.2 (*c* 0.03, MeOH); CD (MeOH) 242 (Δ*ε* +1.86), 283 (Δ*ε* −0.62) nm; IR *ν*_max_: 3370, 2920, 2851, 1579, 1543, 1467, 1261, 1156, 1116, 1041, 799, 721 cm^−1^; ^1^H-NMR data, see Table 1; ^13^C-NMR data, see Table 2; HRESI-MS: *m*/*z* 331.1178 [M + H]^+^ (calcd. for C_18_H_19_O_6_, 331.1182), 353.1000 [M + Na]^+^ (calcd. for C_18_H_18_O_6_Na, 353.1001), 369.0738 [M + K]^+^ (calcd. for C_18_H_18_O_6_K, 369.0740). The original HRESIMS, IR, and 1D- and 2D-NMR spectra of **2** were shown in Supplementary Materials.

*(3S)-7,2′-Dihydroxy-8,4′-dimethoxyisoflavane* (**3**): White amorphous powder, [α]${}_{D}^{25}$ = −7.9 (*c* 0.05, MeOH); CD (MeOH) 234 (Δ*ε* +2.43), 284 (Δ*ε* −0.50) nm; IR *ν*_max_: 3443, 2921, 2851, 1617, 1595, 1463, 1242, 1200, 1165, 1088, 1040, 960, 840, 794, 720 cm^−1^; ^1^H-NMR data, see Table 1; ^13^C-NMR data, see Table 2; HRESI-MS: *m*/*z* 325.1052 [M + Na]^+^ (calcd. for C_17_H_18_O_5_Na, 325.1052). The original HRESIMS, IR, and 1D- and 2D-NMR spectra of **3** were shown in Supplementary Materials.

*7,2′-Dihydroxy-8-methoxy-4′,5′-methylenedioxyisoflavan-3-en-2-one* (**5**): Yellow powder, IR *ν*_max_: 3257, 2921, 2851, 1711, 1601, 1505, 1463, 1359, 1317, 1239, 1203, 1087, 1033, 975, 857, 803 cm^−1^; ^1^H-NMR data, see Table 1; ^13^C-NMR data, see Table 2; HRESI-MS: *m*/*z* 351.0473 [M + Na]^+^ (calcd. for C_17_H_12_O_7_Na, 351.0481). The original HRESIMS, IR, and 1D- and 2D-NMR spectra of **5** were shown in Supplementary Materials.

### 3.4. Cytotoxic Activity Assay

The cytotoxic activity of the isolates against the MCF-7 and MDA-MB-231 human breast cancer cells were determined by a colorimetric MTT assay, as described in the literature [9,28]. Paclitaxel was used as a positive control. The IC_50_ values represented the mean of three independent replicates.

## 4. Conclusions

*Spatholobus suberectus* mainly contains flavonoids, including flavones, flavanones, chalcones, isoflavanes, and isoflavones, some of which have a cytotoxic effect against breast cancer cell lines. In this study, four novel isoflavanes (**1**–**3** and **5**) and four known analogues (**4** and **6**–**8**) were isolated from the rattans of *S. suberectus*. Compounds **1**, **2**, and **6** showed selective cytotoxicity against the estrogen-dependent breast cancer cell line (MCF-7) and the estrogen-independent breast cancer cell line (MDA-MB-231) (**1** and **2** against MCF-7; **6** against MDA-MB-231), while compound **4** had cytotoxicity against both cell lines. Preliminary analysis of the structure–cytotoxicity relationship suggested that C-2′ and C-8 substituents may affect the cytotoxicity and selectivity.

## Figures and Tables

**Table 1:** ^1^H-NMR (600 MHz) data of **1**–**3** and **5** in CDCl_3_ (*δ* in ppm, *J* in Hz) ^a^.

No.	1	2	3	5
2a	4.40 ddd (10.2, 3.6, 1.8)	4.39 ddd (10.2, 2.4, 1.2)	4.44 ddd (10.2, 3.6, 1.8)	
2b	4.03 t (10.2)	4.02 t (10.2)	4.07 t (10.2)	
3	3.56 m	3.60 m	3.51 m	
4a	2.98 ddd (15.6, 11.4, 0.6)	2.94 dd (15.6, 10.2)	3.02 ddd (15.6, 10.8, 1.2)	7.79 s
4b	2.86 ddd (15.6, 4.8, 1.2)	2.88 dd (15.6, 4.8)	2.90 ddd (15.6, 4.8, 1.2)	
5	6.71 d (8.4)	6.73 d (8.4)	6.72 brd (8.4)	7.23 d (8.4)
6	6.51 d (8.4)	6.53 d (8.4)	6.52 d (8.4)	7.00 d (8.4)
3′	6.49 d (2.4)	6.58 s	6.36 d (2.4)	6.61 s
5′	6.46 dd (8.4, 2.4)		6.48 dd (8.4, 2.4)	
6′	7.02 d (8.4)	6.66 s	7.02 d (8.4)	6.72 s
OH-7	5.63 s	5.66 s	5.63 s	6.25 s
OH-2′			4.88 brs	7.42 s
OMe-8	3.91 s	3.93 s	3.91 s	4.18 s
OMe-2′	3.82 s	3.81 s		
OMe-4′	3.80 s		3.77 s	
OCH_2_O		5.90 s		5.97 s

^a^ The assignments were based on ^1^H–^1^H COSY, HSQC, and HMBC experiments.

**Table 2:** ^13^C-NMR (125 MHz) data of **1**–**3** and **5** in CDCl_3_ (*δ* in ppm).

No.	1	2	3	5
2	70.3	70.3	69.9	162.8
3	31.6	31.7	31.6	123.4
4	30.7	30.8	30.4	144.2
5	124.4	124.4	124.2	123.7
6	107.0	107.1	106.9	113.4
7	147.6	147.7	147.4	152.2
8	135.0	135.0	134.8	133.5
9	147.4	147.3	147.1	146.0
10	115.7	115.4	115.3	114.2
1′	121.8	121.6	119.7	115.2
2′	158.4	152.5	154.2	150.7
3′	98.9	95.0	102.1	101.6
4′	159.9	146.9	159.4	149.8
5′	104.3	141.4	106.0	142.6
6′	127.7	107.2	128.2	108.9
OMe-8	61.1	61.0	60.9	62.1
OMe-2′	55.5	56.6		
OMe-4′	55.5		55.3	
OCH_2_O		101.3		101.8

^a^ The assignments were based on ^1^H–^1^H COSY, HSQC, and HMBC experiments.

**Table 3:** Cytotoxicity against MCF-7 and MDA-MB-231 cells of **1**–**8**.

No.	IC_50_ (μM)	
	MCF-7	MDA-MB-231
**1**	59.0 ± 8.1	>100
**2**	93.6 ± 17.3	>100
**3**	>100	>100
**4**	60.1 ± 7.4	34.1 ± 6.3
**5**	>100	>100
**6**	>100	25.1 ± 7.7
**7**	>100	>100
**8**	>100	>100

## Supplementary Materials

The following are available online, Figures S1–S31: HRESIMS, IR, and 1D- and 2D-NMR spectra of new compounds **1**–**3** and **5**.
