# Supplementary material for: New Isoflavanes from Spatholobus suberectus and Their Cytotoxicity against Human Breast Cancer Cell Lines
Source: Molecules. 2019 Sep 4;24(18):3218. doi: 10.3390/molecules24183218 (PMC6766798; doi:10.3390/molecules24183218)
Supplement: Supplementary file 1 [file molecules-24-03218-s001.pdf]

molecules

Article

New Isoflavanes from *Spatholobus suberectus* and Their Cytotoxicity against Human Breast Cancer Cell Lines

Fu Peng [1], Huan Zhu [2,3], Chun-Wang Meng [2,3], Yan-Rui Ren [2], Ou Dai [2,3,*] and Liang Xiong [2,3,*]

[1] West China School of Pharmacy, Sichuan University, Chengdu 610041, China; pengf@scu.edu.cn (F.P.)
[2] School of Pharmacy, Chengdu University of Traditional Chinese Medicine, Chengdu 611137, China; 18482134471@163.com (H.Z.); mengchunw@126.com (C.-W.M.); ryr08094523@163.com (Y.-R.R.)
[3] Institute of Innovative Medicine Ingredients of Southwest Specialty Medicinal Materials, Chengdu University of Traditional Chinese Medicine, Chengdu 611137, China
* Correspondence: oudai1123@hotmail.com (O.D.); xiling@cdutcm.edu.cn (L.X.)

The List of Contents

Content	Page
**Figure S1.** The (+)-HRESIMS Spectroscopic Data of Compound **1**	S1
**Figure S2.** The IR Spectrum of Compound **1**	S2
**Figure S3.** The ^{1}H NMR Spectrum of Compound **1** in $CDCl_3$	S3
**Figure S4.** The ^{13}C NMR Spectrum of Compound **1** in $CDCl_3$	S4
**Figure S5.** The DEPT Spectrum of Compound **1** in $CDCl_3$	S5
**Figure S6.** The HSQC Spectrum of Compound **1** in $CDCl_3$	S6
**Figure S7.** The ^{1}H-^{1}H gCOSY Spectrum of Compound **1** in $CDCl_3$	S7
**Figure S8.** The HMBC Spectrum of Compound **1** in $CDCl_3$	S8
**Figure S9.** The (+)-HRESIMS Spectroscopic Data of Compound **2**	S9
**Figure S10.** The IR Spectrum of Compound **2**	S10
**Figure S11.** The ^{1}H NMR Spectrum of Compound **2** in $CDCl_3$	S11
**Figure S12.** The ^{13}C NMR Spectrum of Compound **2** in $CDCl_3$	S12
**Figure S13.** The DEPT Spectrum of Compound **2** in $CDCl_3$	S13
**Figure S14.** The HSQC Spectrum of Compound **2** in $CDCl_3$	S14
**Figure S15.** The ^{1}H-^{1}H gCOSY Spectrum of Compound **2** in $CDCl_3$	S15
**Figure S16.** The HMBC Spectrum of Compound **2** in $CDCl_3$	S16
**Figure S17.** The (+)-HRESIMS Spectroscopic Data of Compound **3**	S17
**Figure S18.** The IR Spectrum of Compound **3**	S18
**Figure S19.** The ^{1}H NMR Spectrum of Compound **3** in $CDCl_3$	S19
**Figure S20.** The ^{13}C NMR Spectrum of Compound **3** in $CDCl_3$	S20
**Figure S21.** The HSQC Spectrum of Compound **3** in $CDCl_3$	S21
**Figure S22.** The ^{1}H-^{1}H gCOSY Spectrum of Compound **3** in $CDCl_3$	S22
**Figure S23.** The HMBC Spectrum of Compound **3** in $CDCl_3$	S23
**Figure S24.** The NOESY Spectrum of Compound **3** in $CDCl_3$	S24
**Figure S25.** The (+)-HRESIMS Spectroscopic Data of Compound **5**	S25
**Figure S26.** The IR Spectrum of Compound **5**	S26
**Figure S27.** The ^{1}H NMR Spectrum of Compound **5** in $CDCl_3$	S27
**Figure S28.** The ^{13}C NMR Spectrum of Compound **5** in $CDCl_3$	S28
**Figure S29.** The HSQC Spectrum of Compound **5** in $CDCl_3$	S29
**Figure S30.** The ^{1}H-^{1}H gCOSY Spectrum of Compound **5** in $CDCl_3$	S30
**Figure S31.** The HMBC Spectrum of Compound **5** in $CDCl_3$	S31

Single Mass Analysis
Tolerance = 2.0 mDa / DBE: min = -1.5, max = 50.0
Element prediction: Off
Number of isotope peaks used for i-FIT = 3
Monoisotopic Mass, Even Electron Ions
126 formula(e) evaluated with 1 results within limits (up to 50 closest results for each mass)
Elements Used:

Mass	Calc. Mass	mDa	PPM	DBE	Formula	i-FIT	i-FIT Norm	Fit Conf %	C	H	O	Na
339.1208	339.1208	0.0	0.0	8.5	C18 H20 O5 Na	827.5	n/a	n/a	18	20	5	1

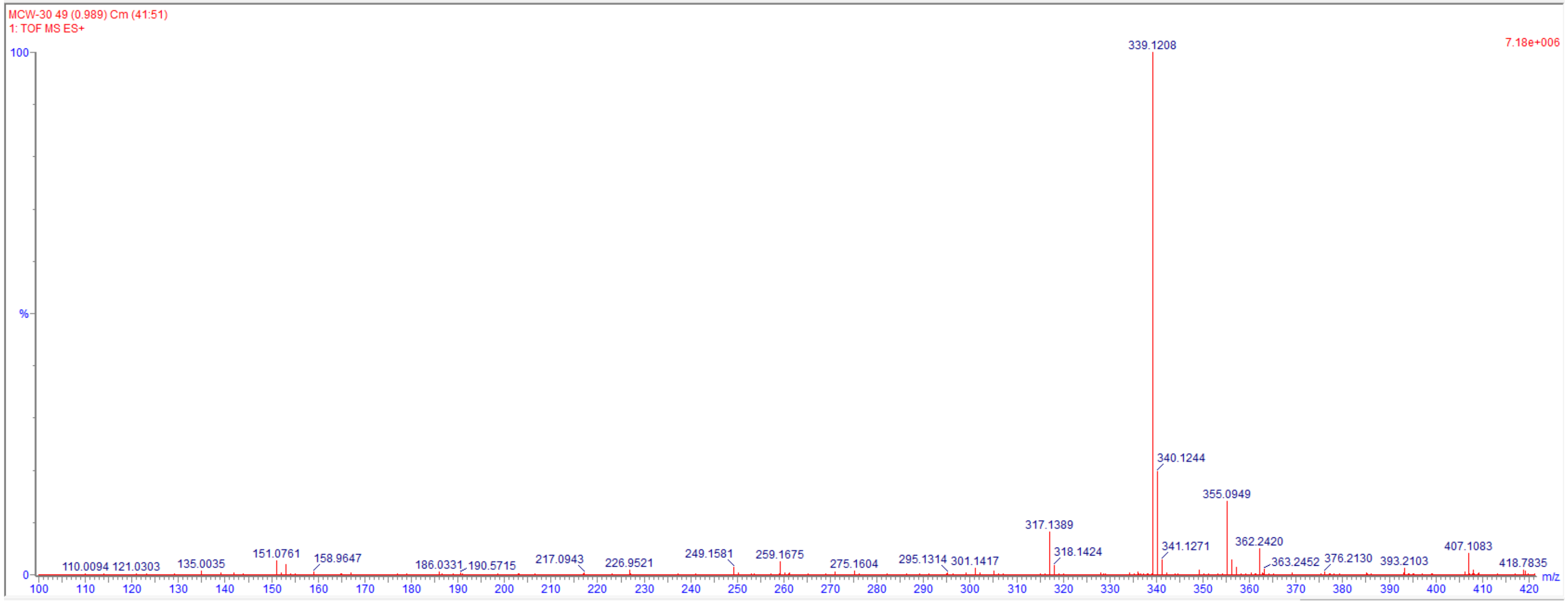

Figure S1. The (+)-HRESIMS Spectroscopic Data of Compound **1**.

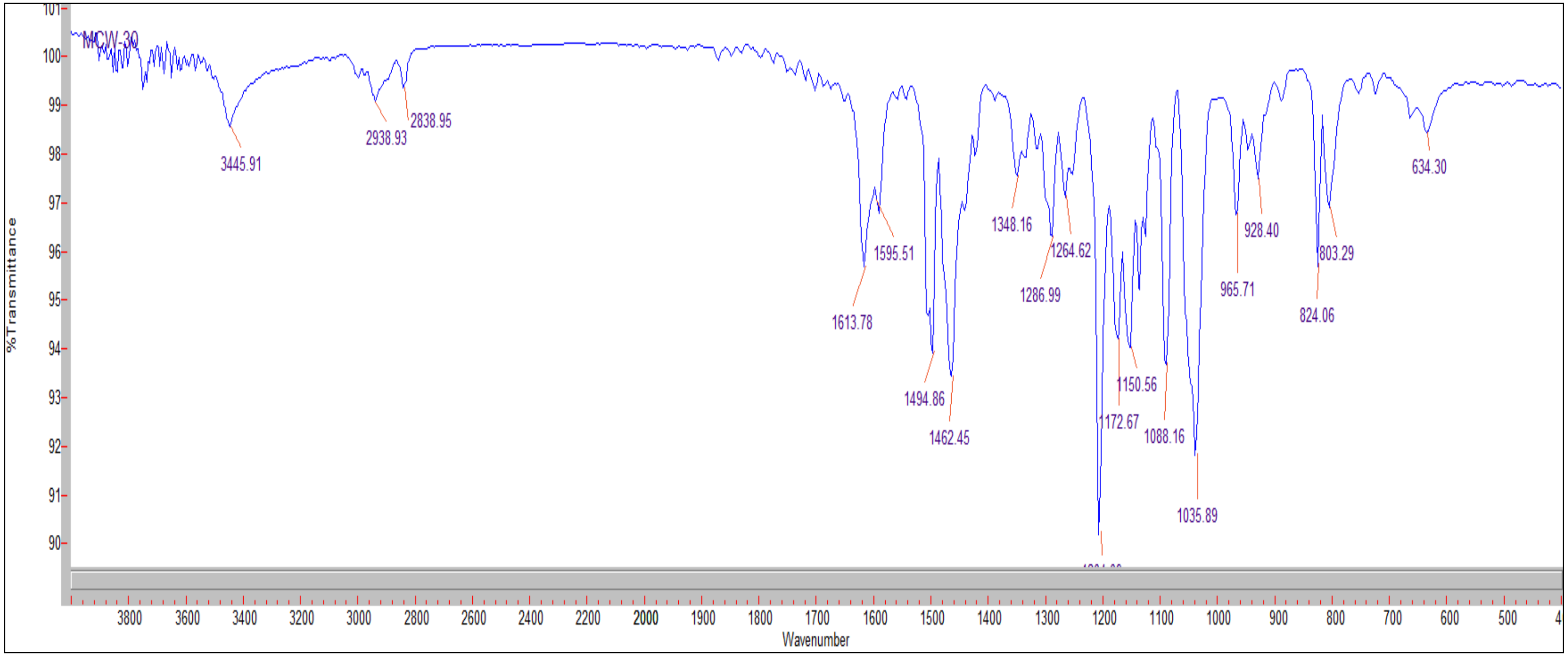

Figure S2. The IR Spectrum of Compound **1**.

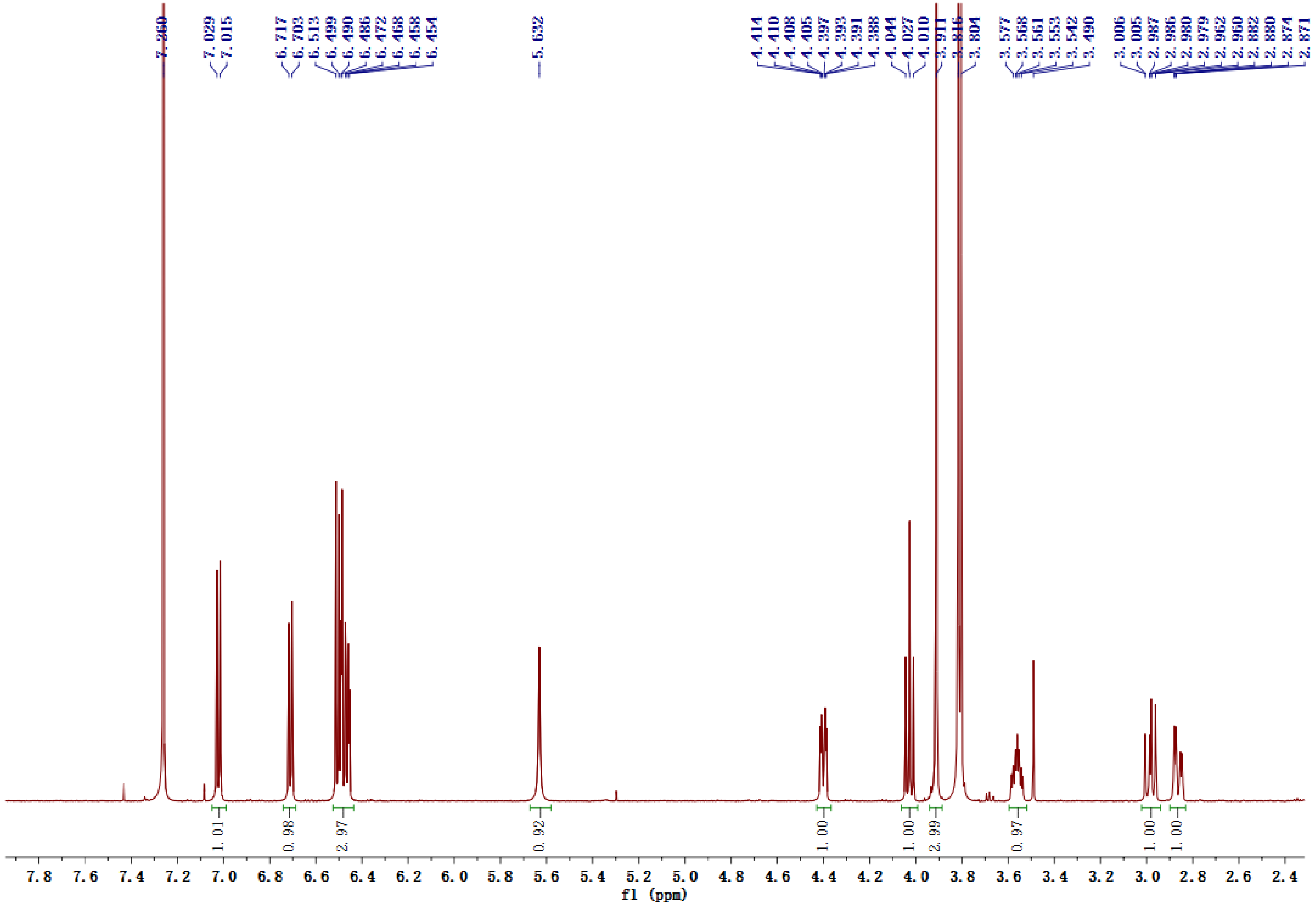

Figure S3. The ^{1}H NMR Spectrum of Compound **1** in $CDCl_3$.

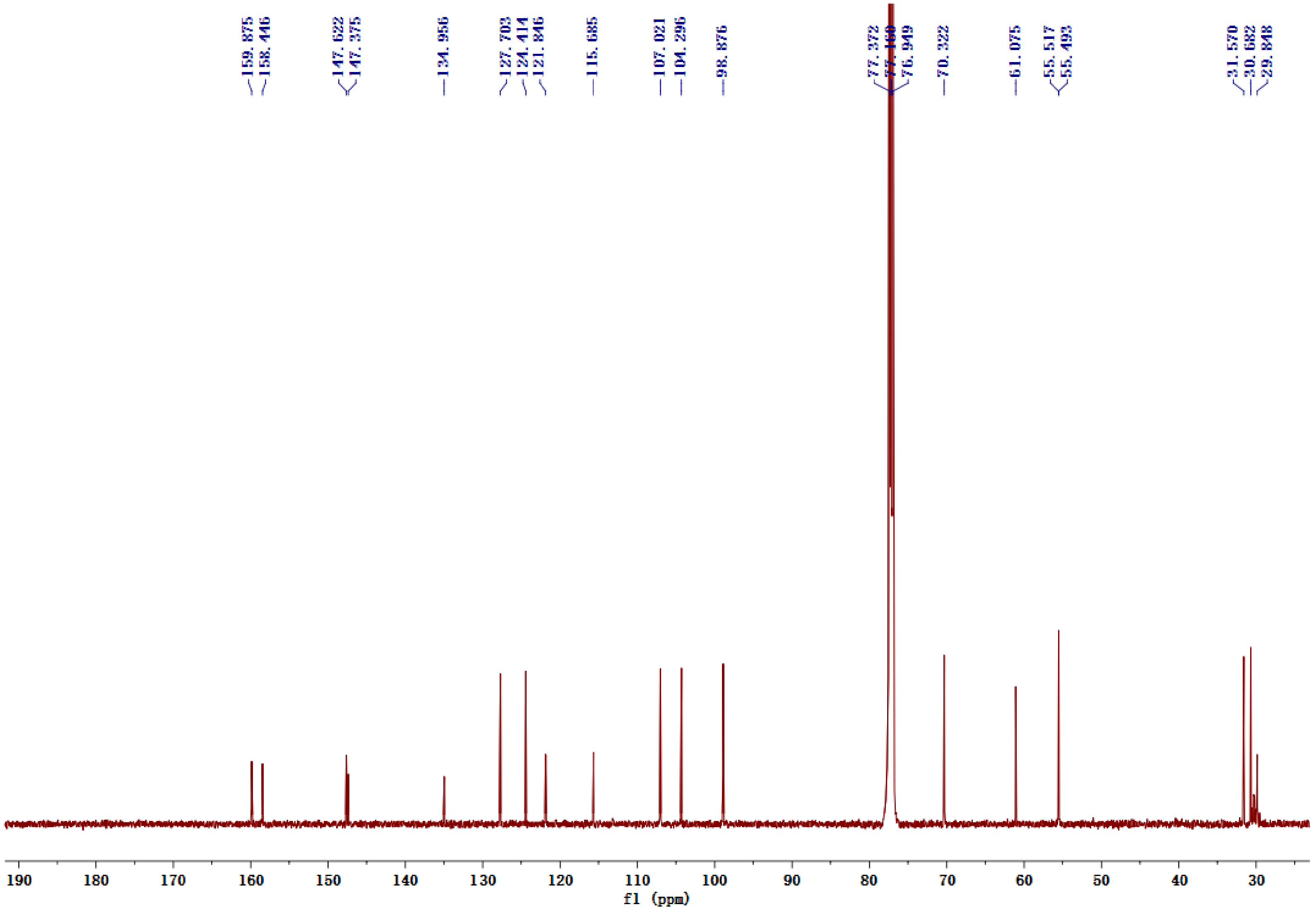

Figure S4. The ^{13}C NMR Spectrum of Compound **1** in $CDCl_3$.

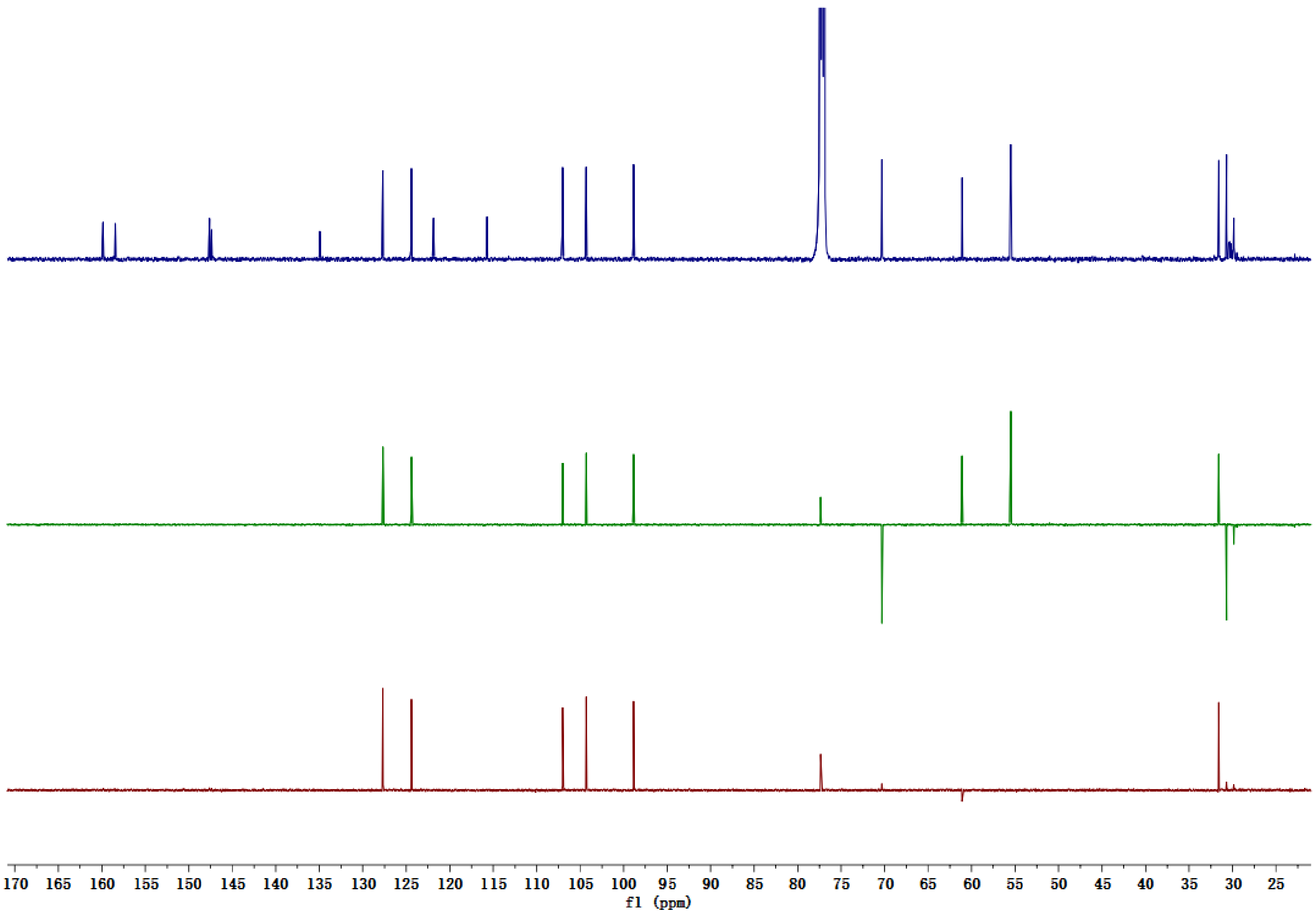

Figure S5. The DEPT Spectrum of Compound **1** in $CDCl_3$.

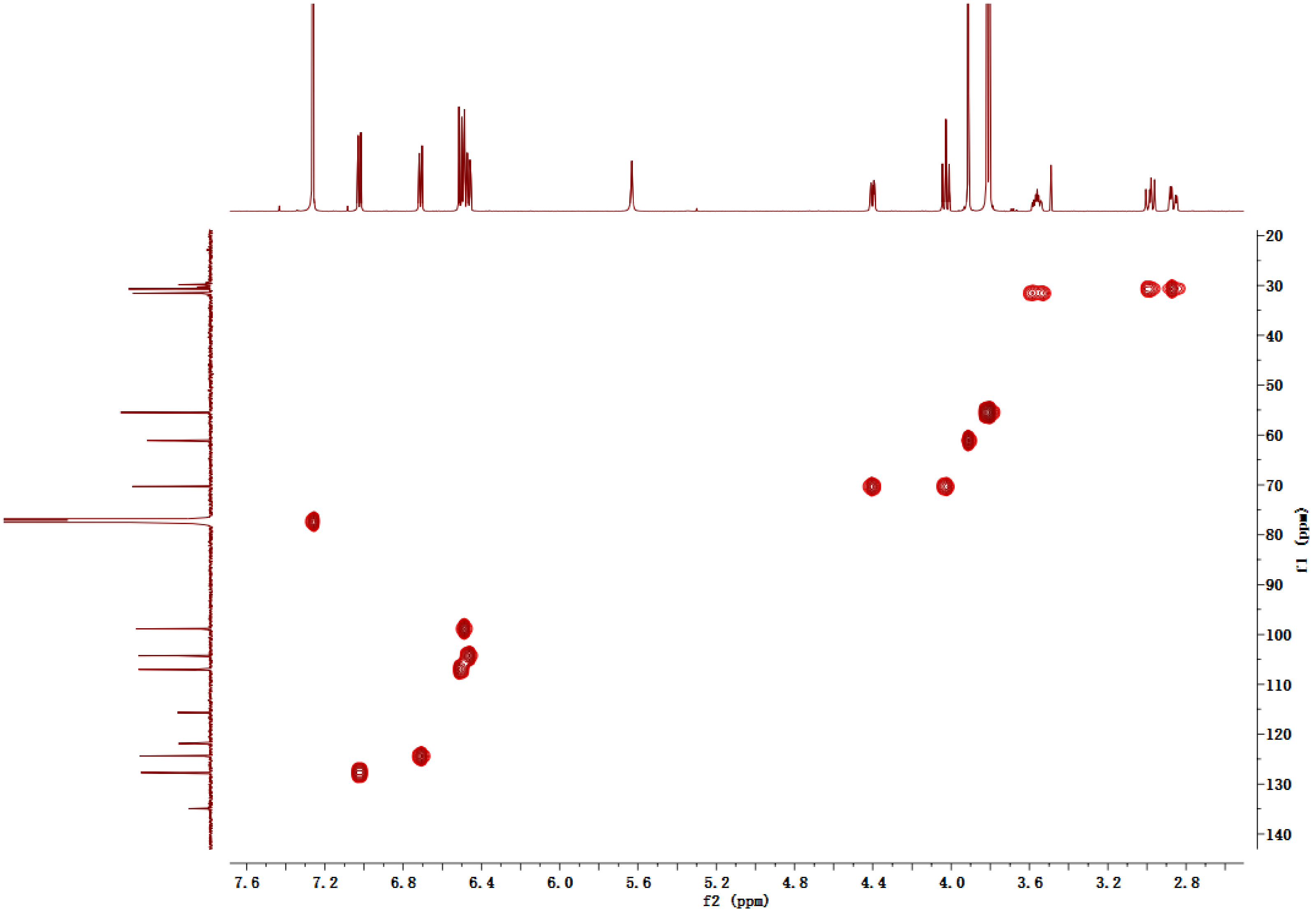

Figure S6. The HSQC Spectrum of Compound **1** in $CDCl_3$.

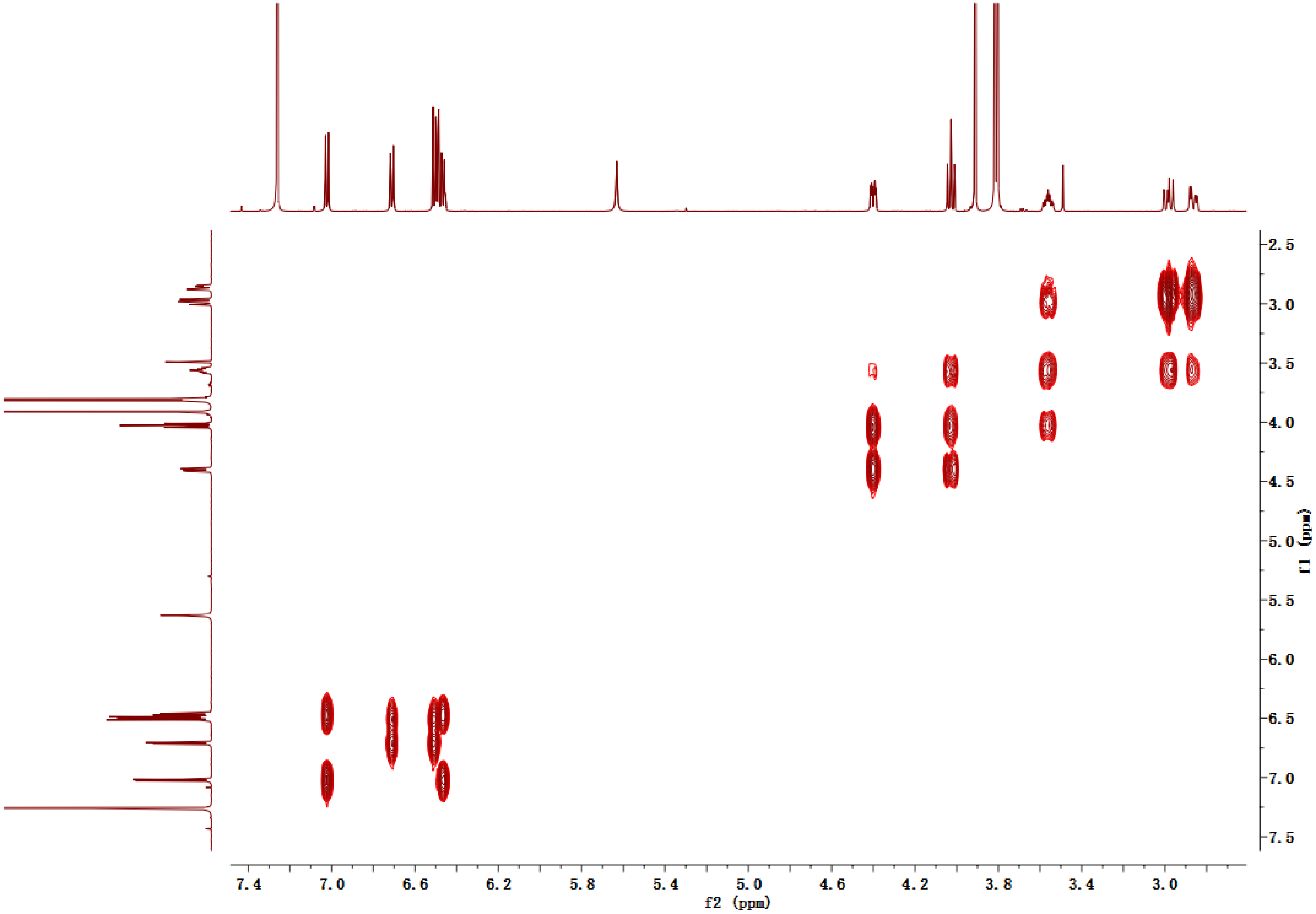

Figure S7. The ^{1}H-^{1}H gCOSY Spectrum of Compound **1** in $CDCl_3$.

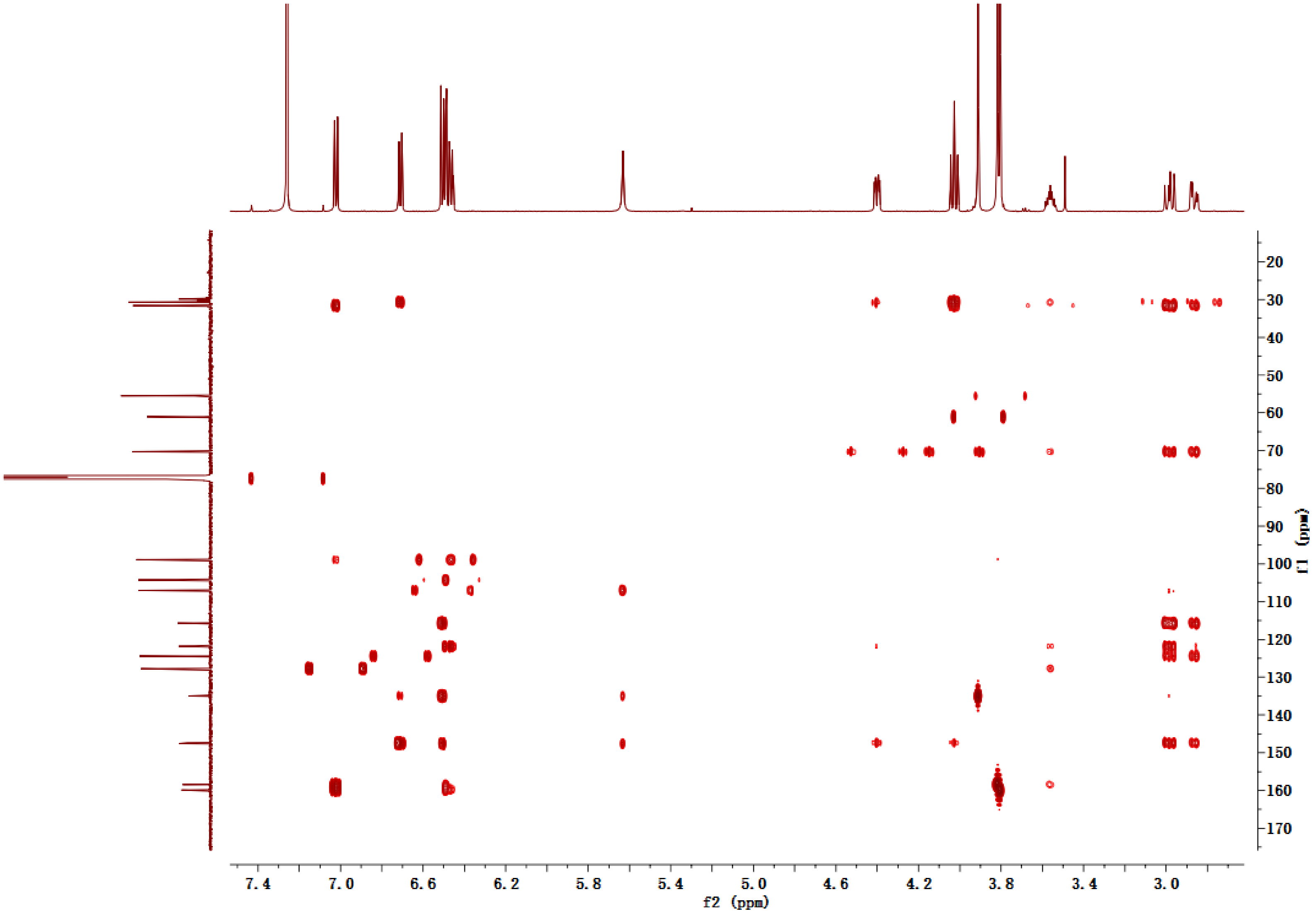

Figure S8. The HMBC Spectrum of Compound **1** in $CDCl_3$.

Single Mass Analysis
Tolerance = 2.0 mDa / DBE: min = -1.5, max = 50.0
Element prediction: Off
Number of isotope peaks used for i-FIT = 3
Monoisotopic Mass, Even Electron Ions
127 formula(e) evaluated with 1 results within limits (up to 50 closest results for each mass)
Elements Used:

Mass	Calc. Mass	mDa	PPM	DBE	Formula	i-FIT	i-FIT Norm	Fit Conf %	C	H	O	Na
353.1000	353.1001	-0.1	-0.3	9.5	C18 H18 O6 Na	733.7	n/a	n/a	18	18	6	1

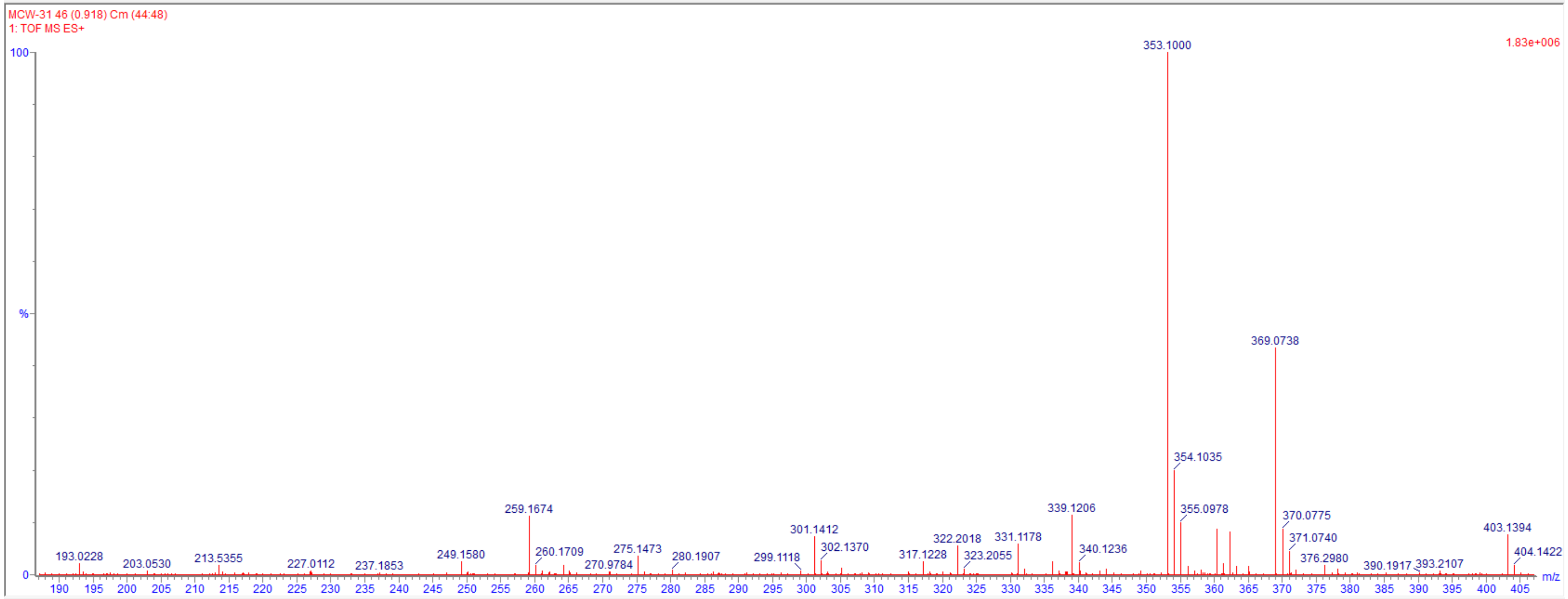

Figure S9. The (+)-HRESIMS Spectroscopic Data of Compound **2**.

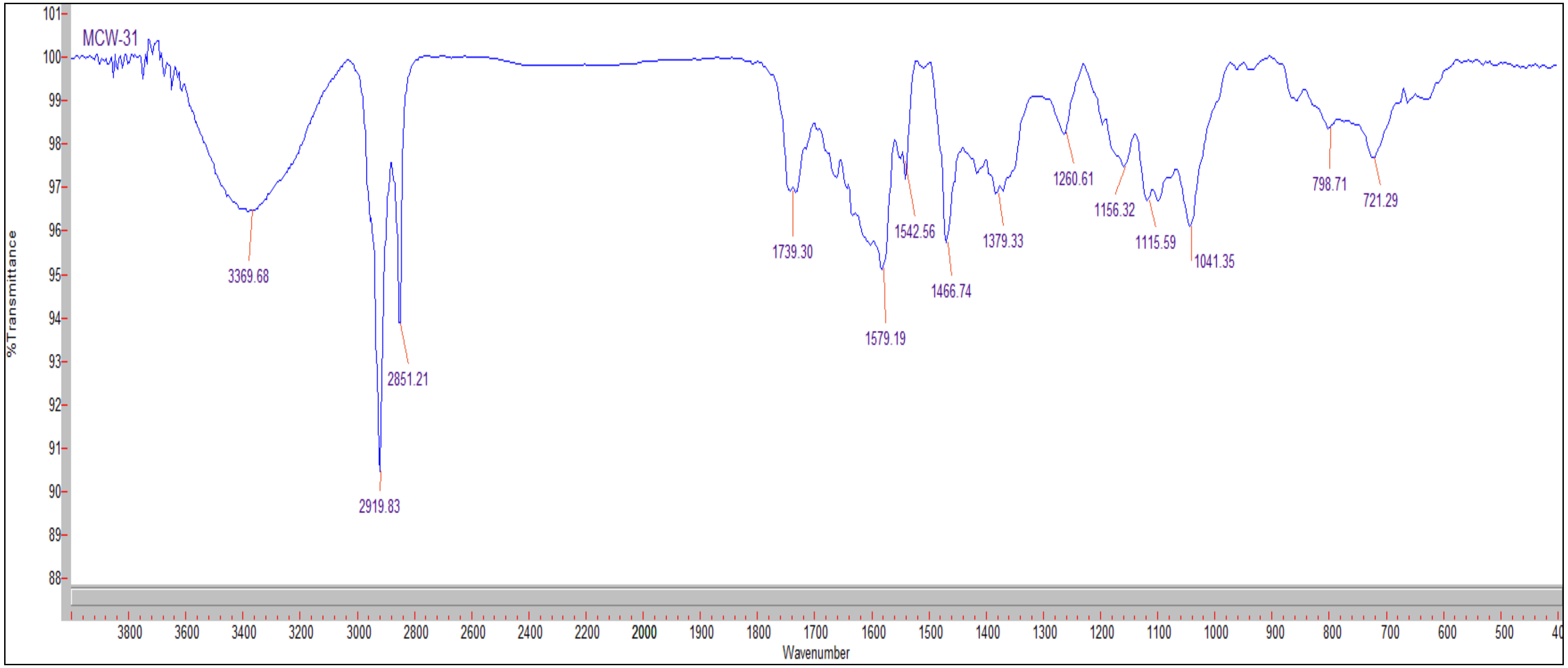

Figure S10. The IR Spectrum of Compound **2**.

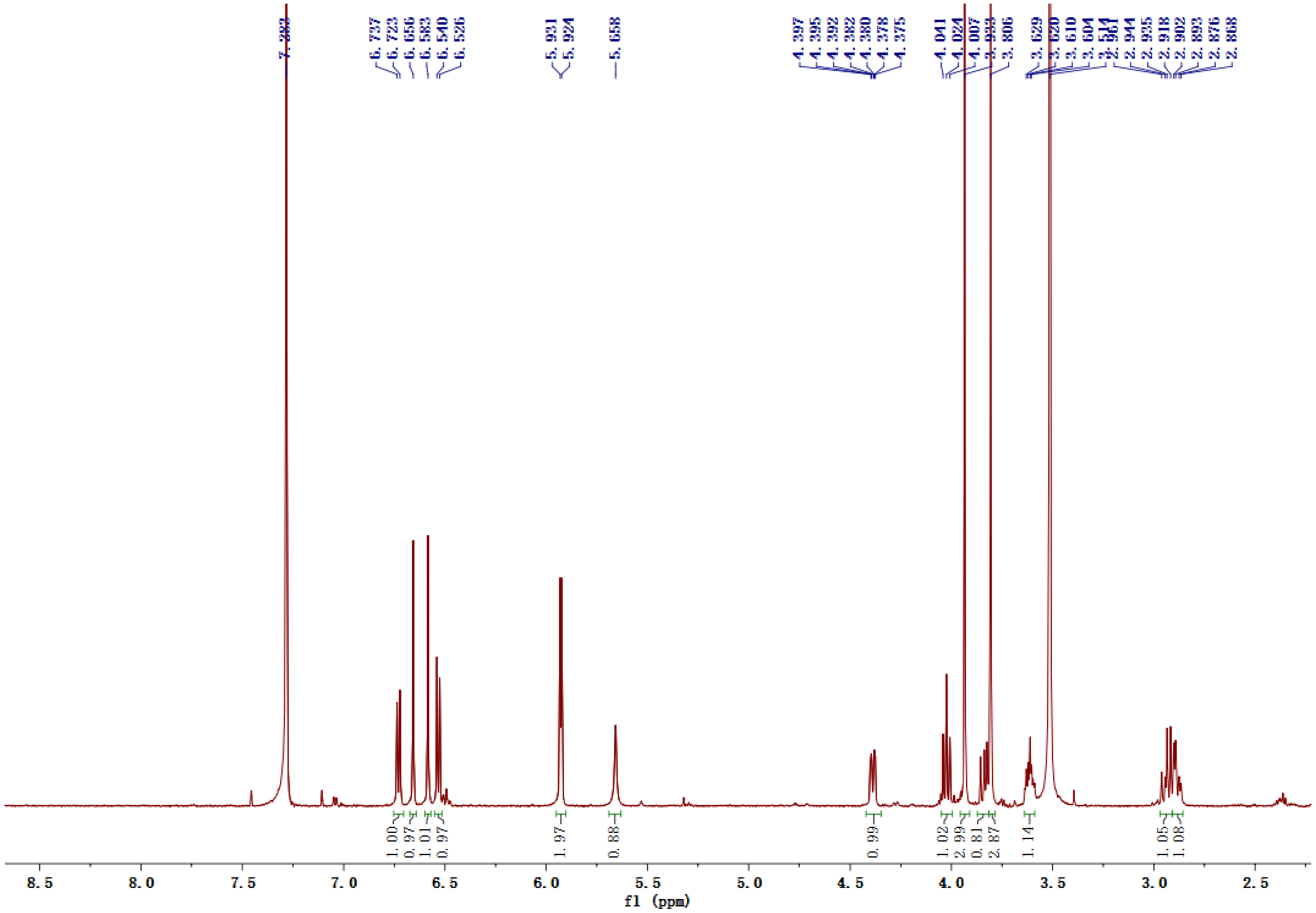

Figure S11. The ^{1}H NMR Spectrum of Compound **2** in $CDCl_3$.

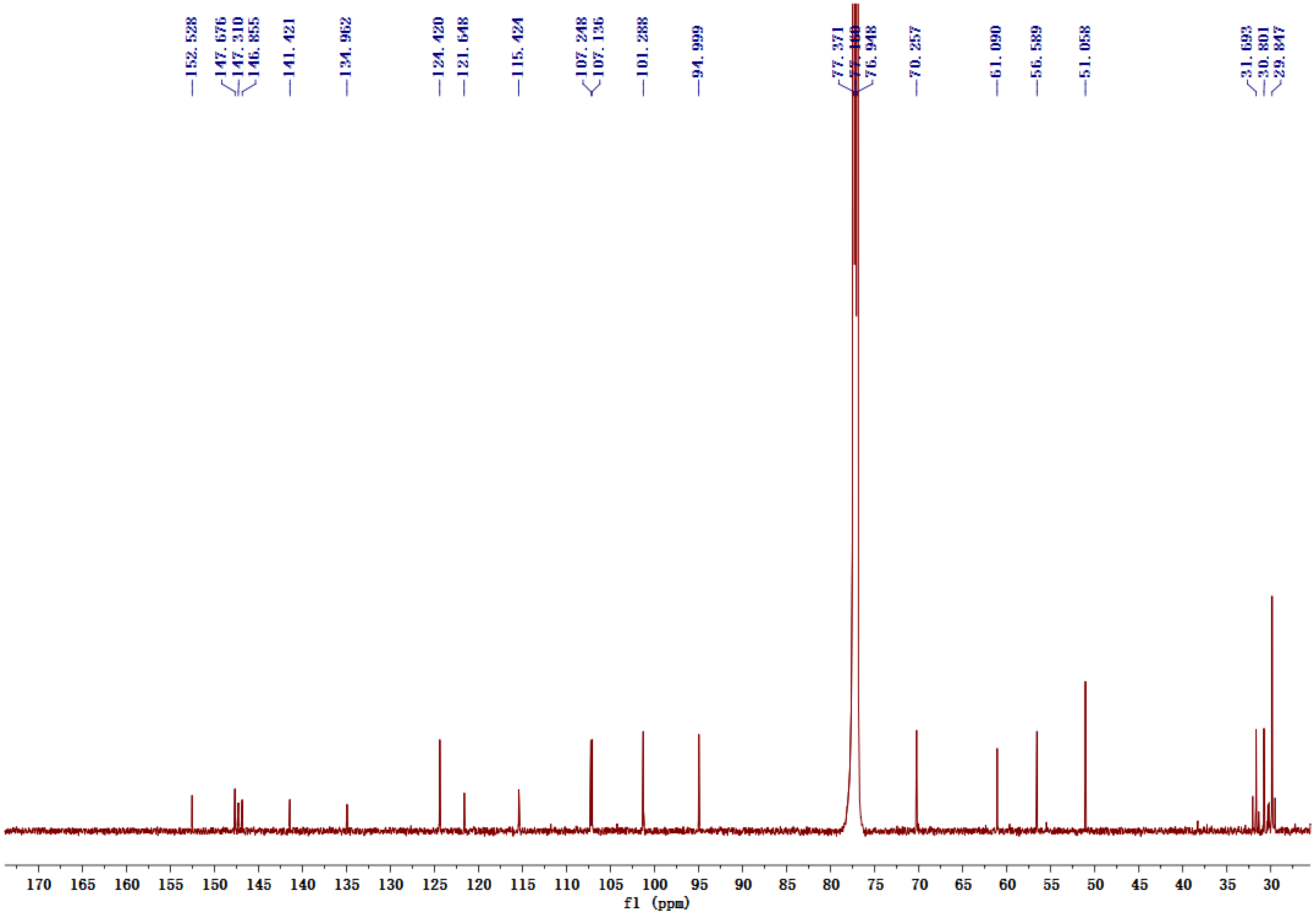

Figure S12. The ^{13}C NMR Spectrum of Compound **2** in $CDCl_3$.

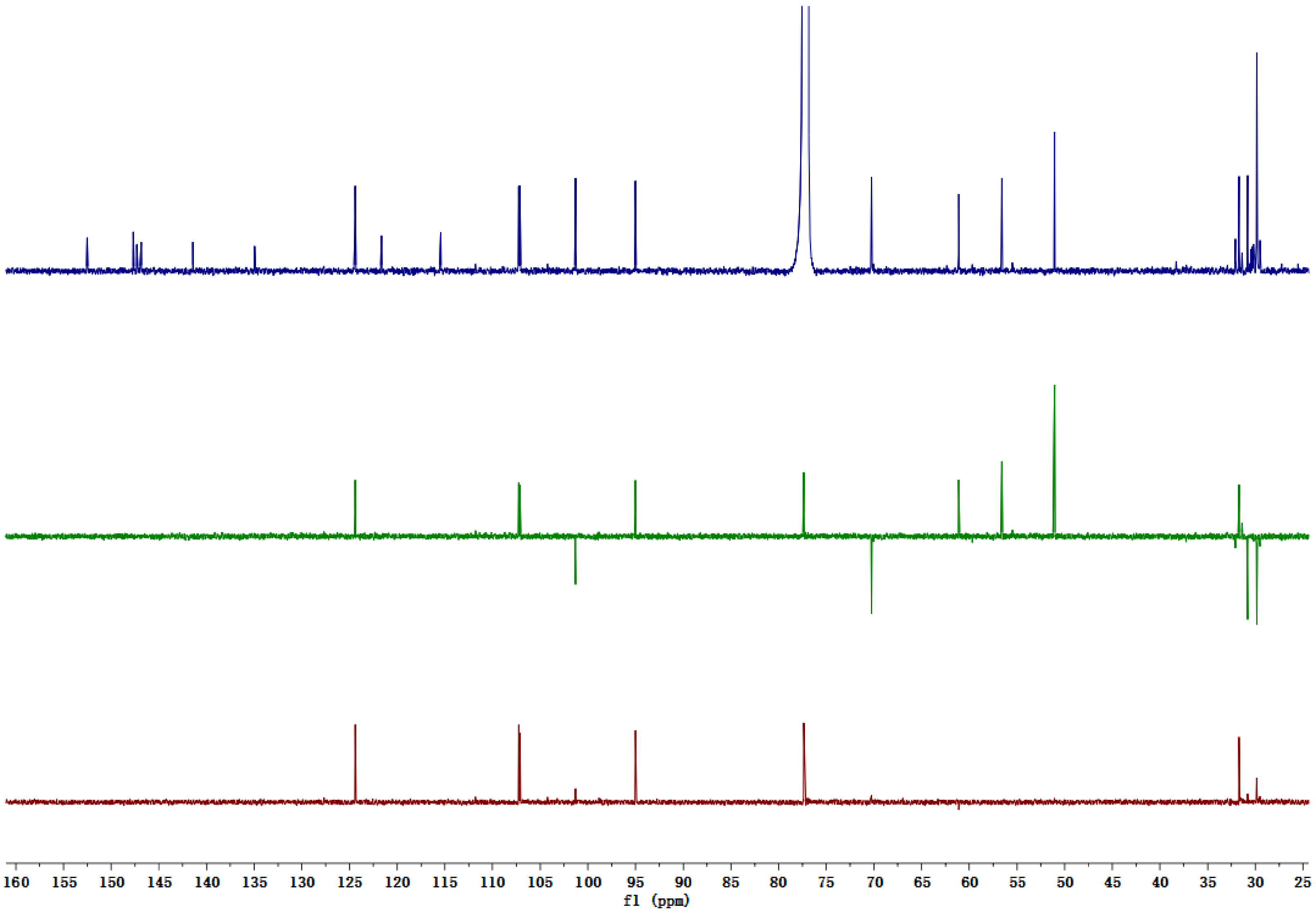

Figure S13. The DEPT Spectrum of Compound **2** in $CDCl_3$.

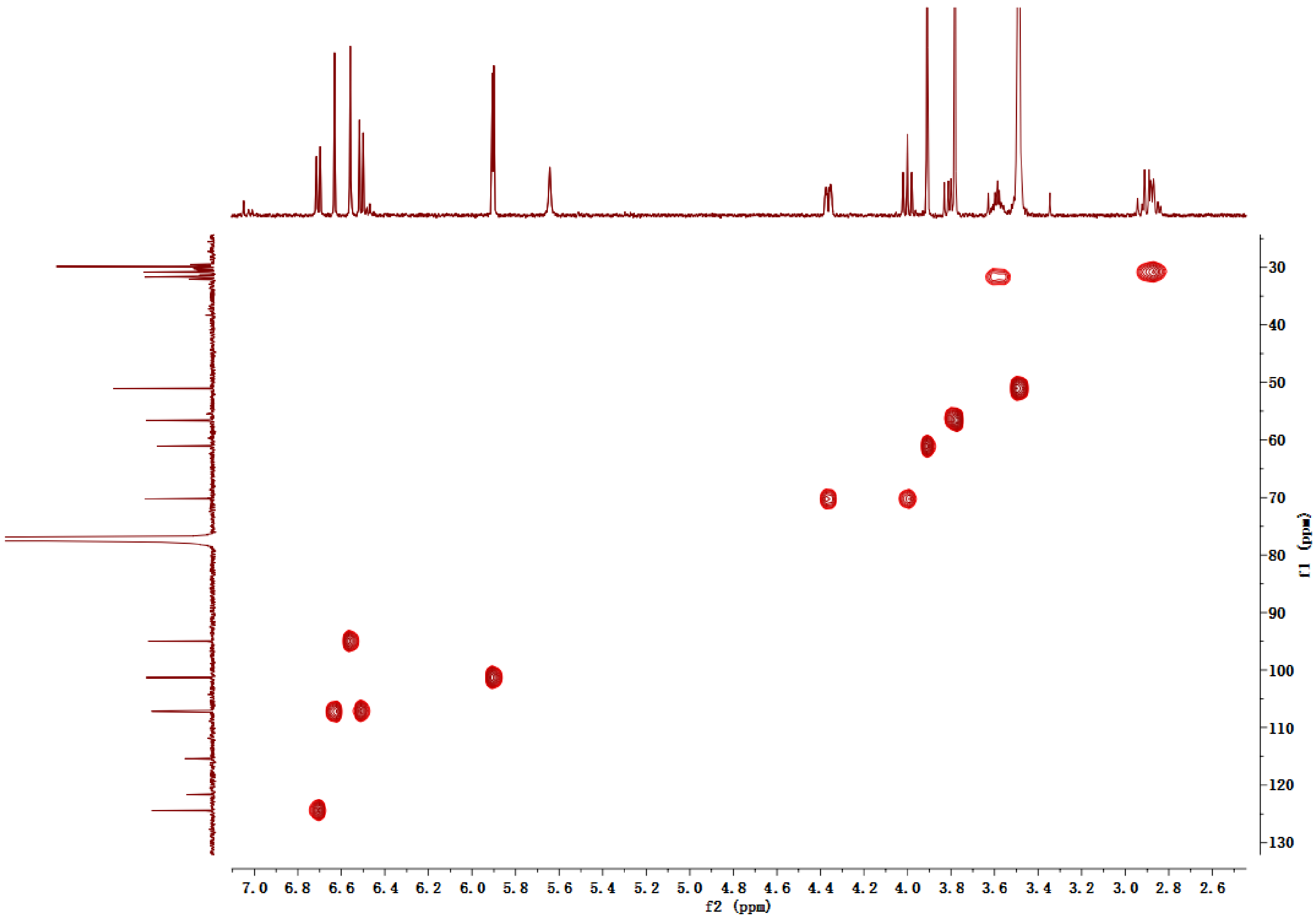

Figure S14. The HSQC Spectrum of Compound **2** in $CDCl_3$.

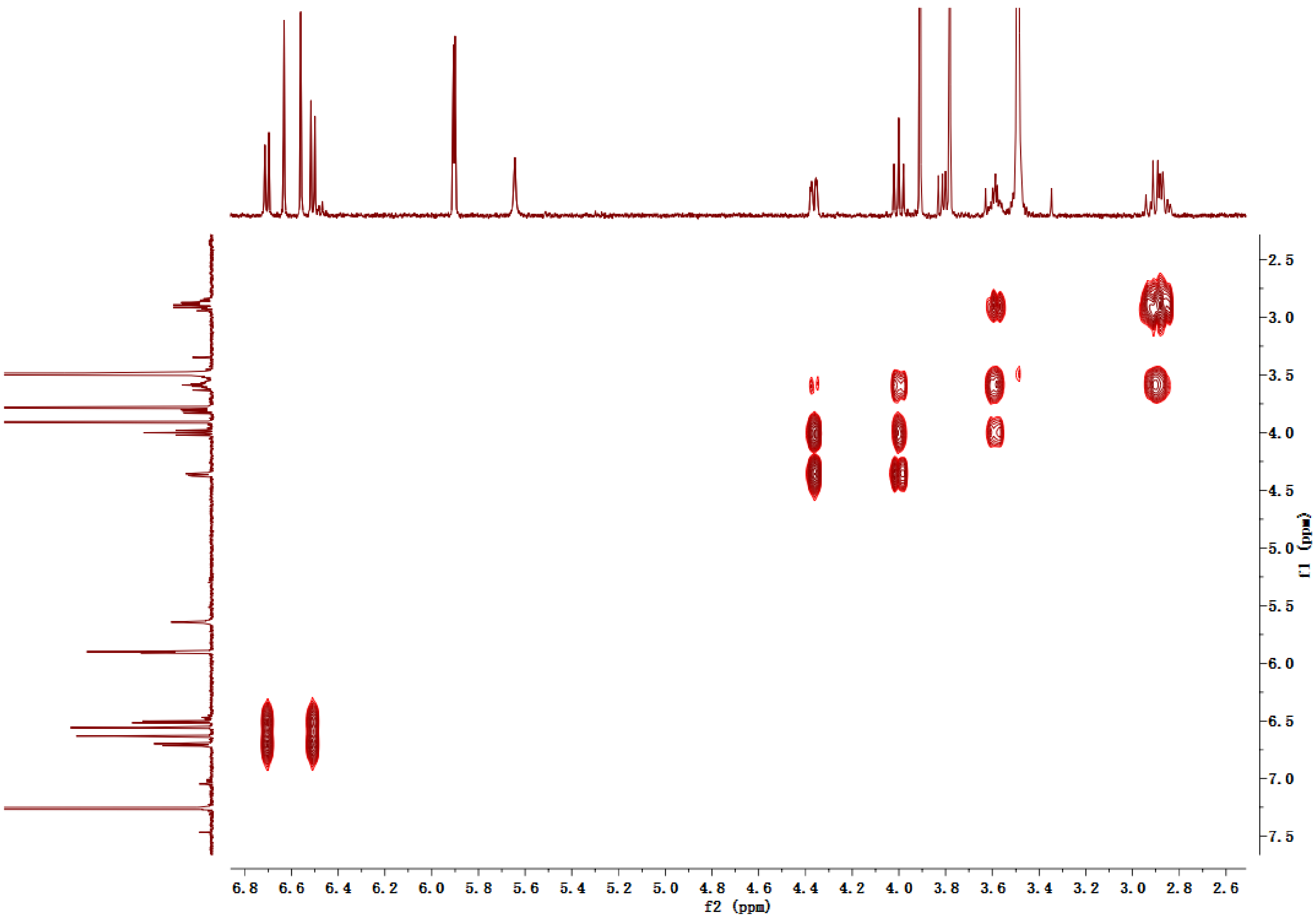

Figure S15. The ^{1}H-^{1}H gCOSY Spectrum of Compound **2** in $CDCl_3$.

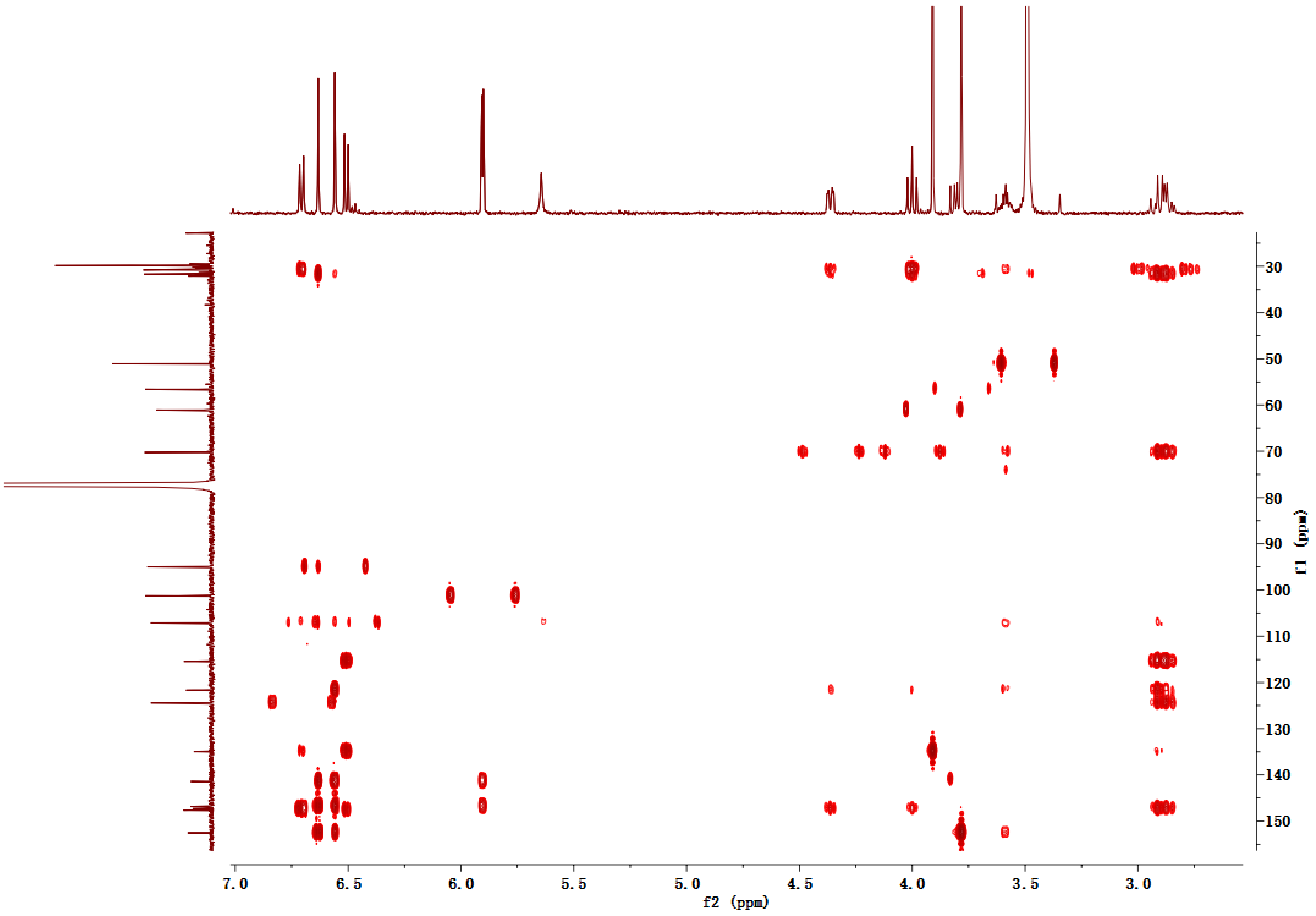

Figure S16. The HMBC Spectrum of Compound **2** in $CDCl_3$.

Single Mass Analysis
Tolerance = 1.0 mDa / DBE: min = -1.5, max = 50.0
Element prediction: Off
Number of isotope peaks used for i-FIT = 5
Monoisotopic Mass, Even Electron Ions
226 formula(e) evaluated with 1 results within limits (up to 50 closest results for each mass)
Elements Used:

Mass	Calc. Mass	mDa	PPM	DBE	Formula	i-FIT	i-FIT Norm	Fit Conf %	C	H	N	O	Na
325.1052	325.1052	0.0	0.0	8.5	C17 H18 O5 Na	1551.7	n/a	n/a	17	18		5	1

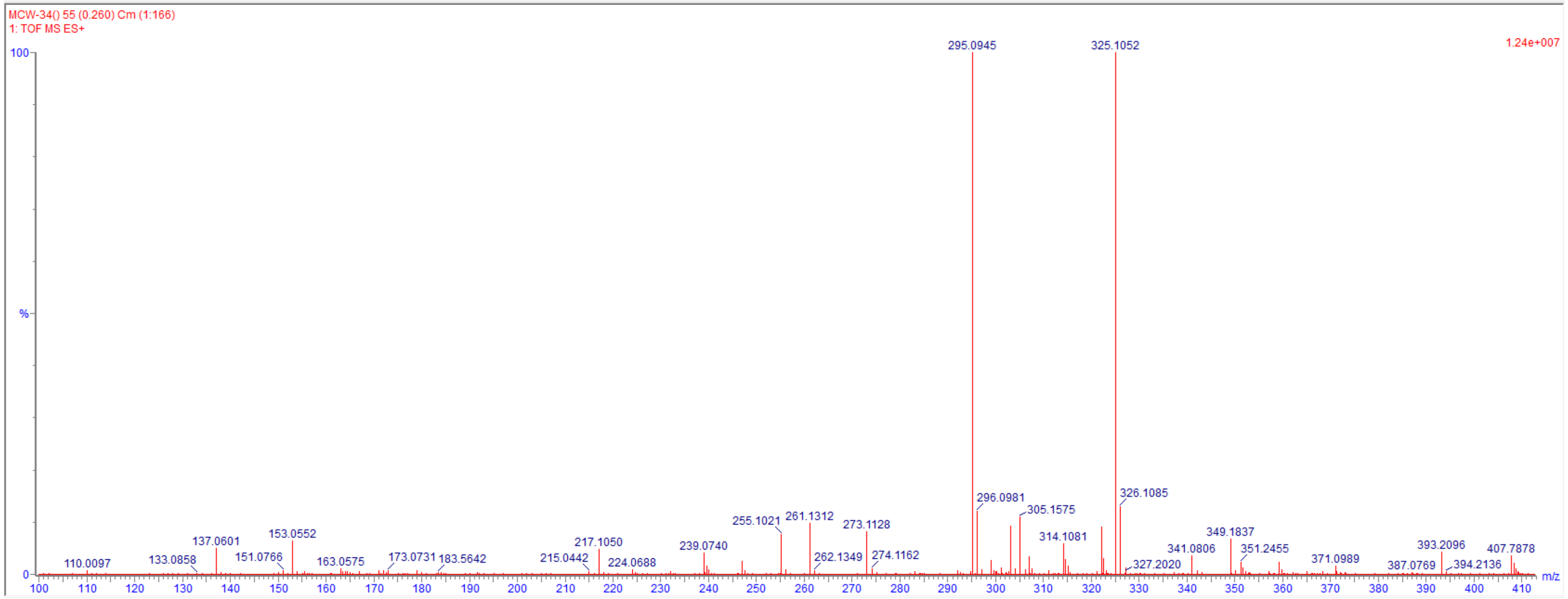

Figure S17. The (+)-HRESIMS Spectroscopic Data of Compound **3.**

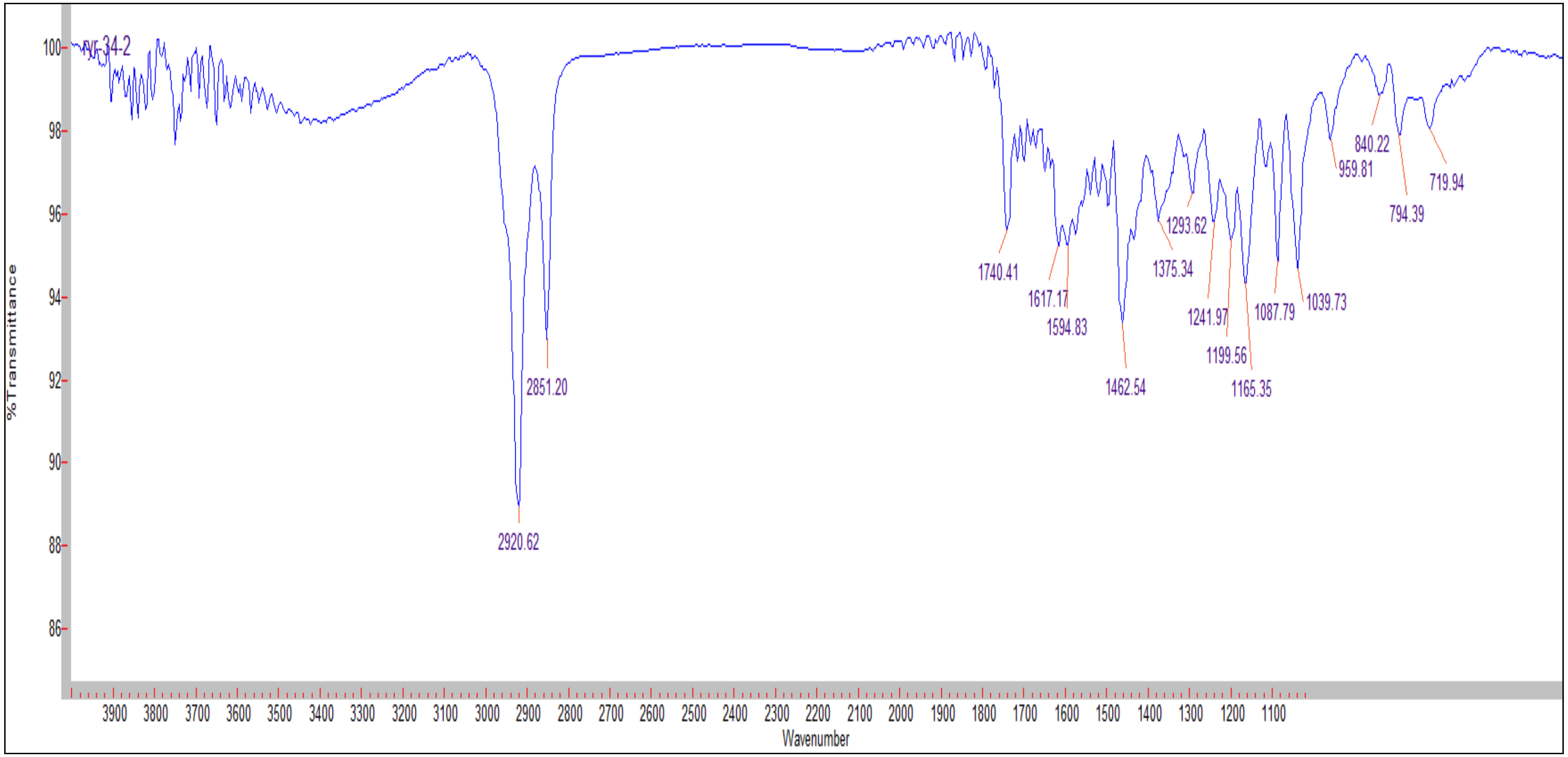

Figure S18. The IR Spectrum of Compound **3**.

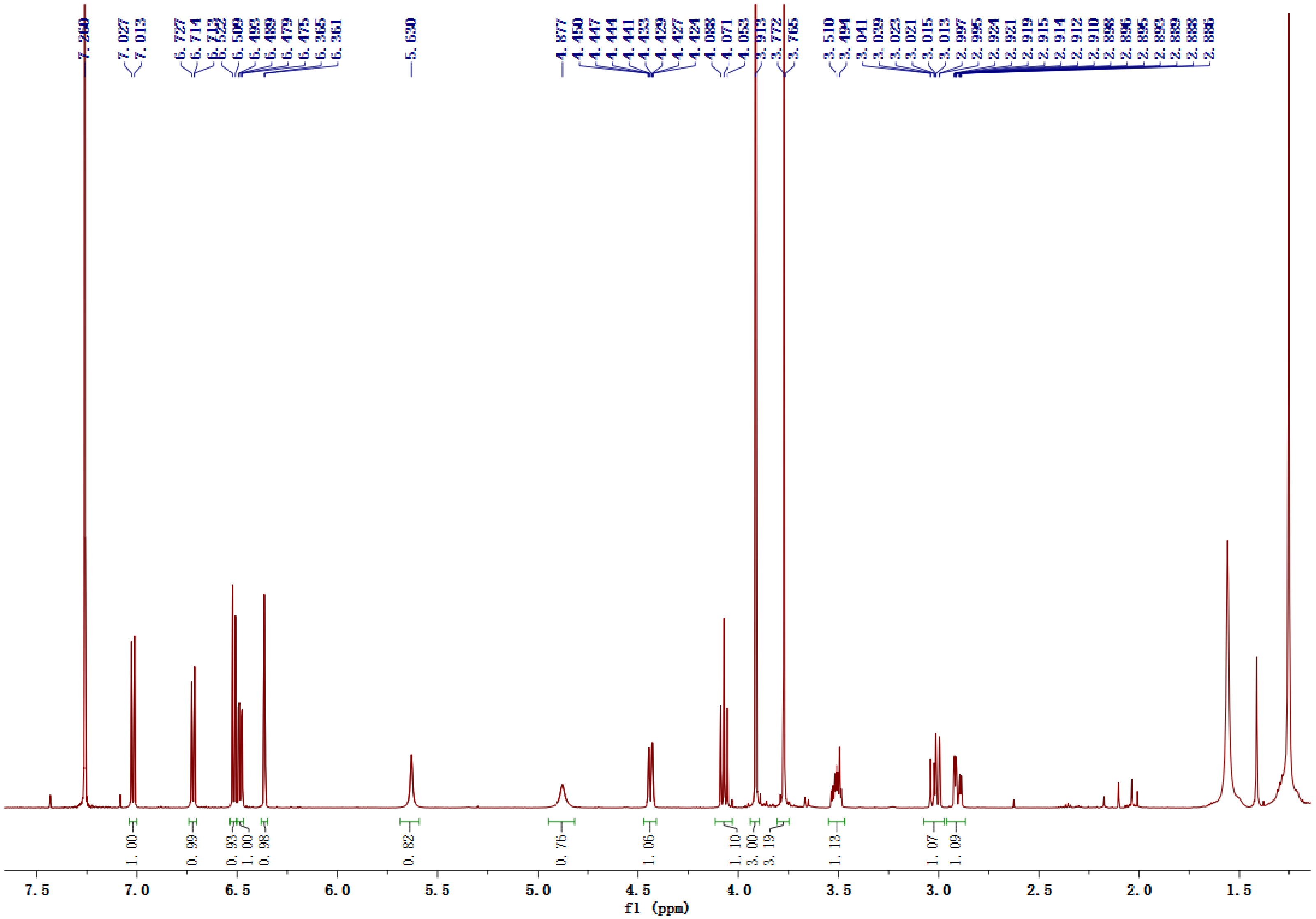

Figure S19. The ^{1}H NMR Spectrum of Compound **3** in $CDCl_3$.

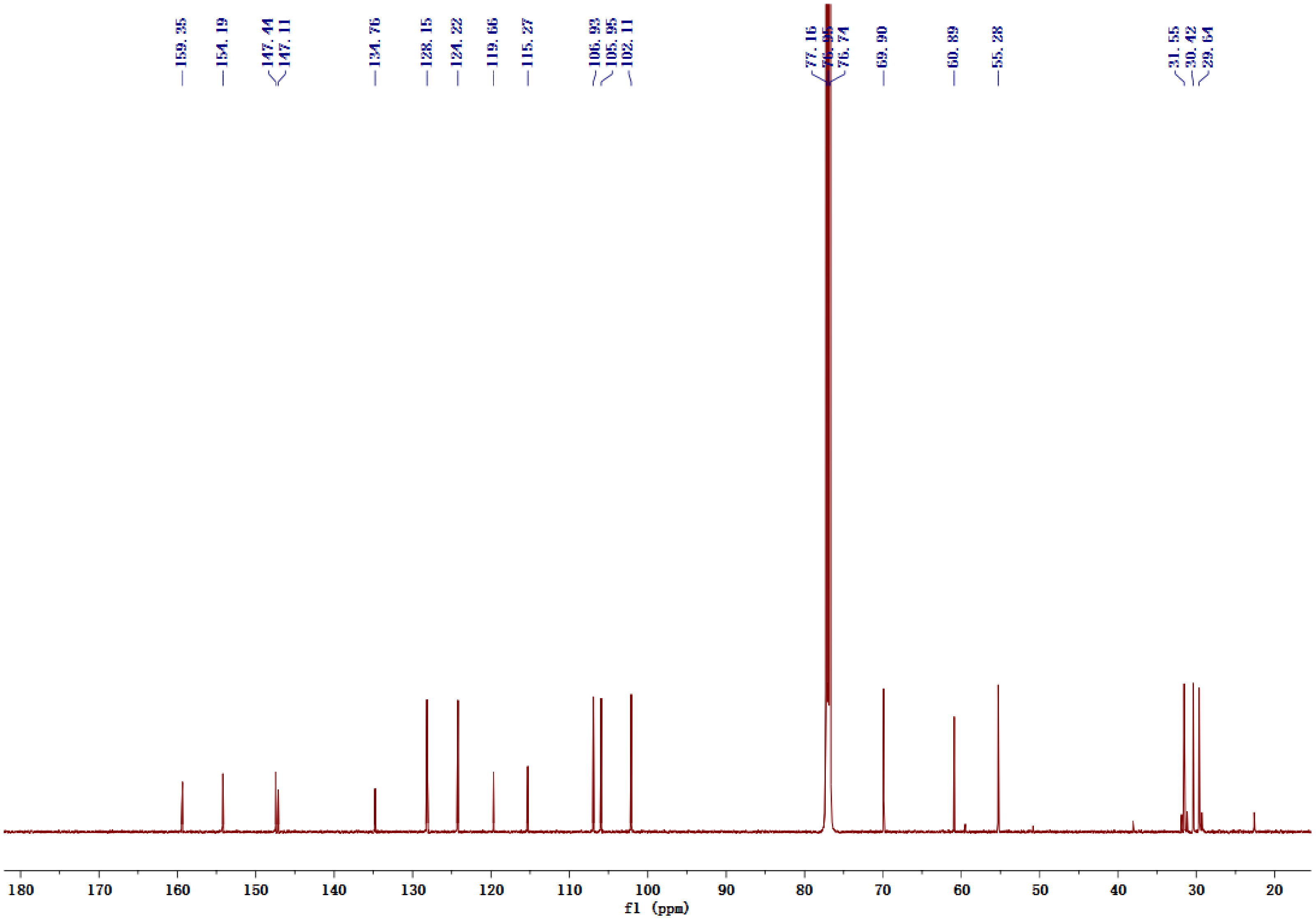

Figure S20. The ^{13}C NMR Spectrum of Compound **3** in $CDCl_3$.

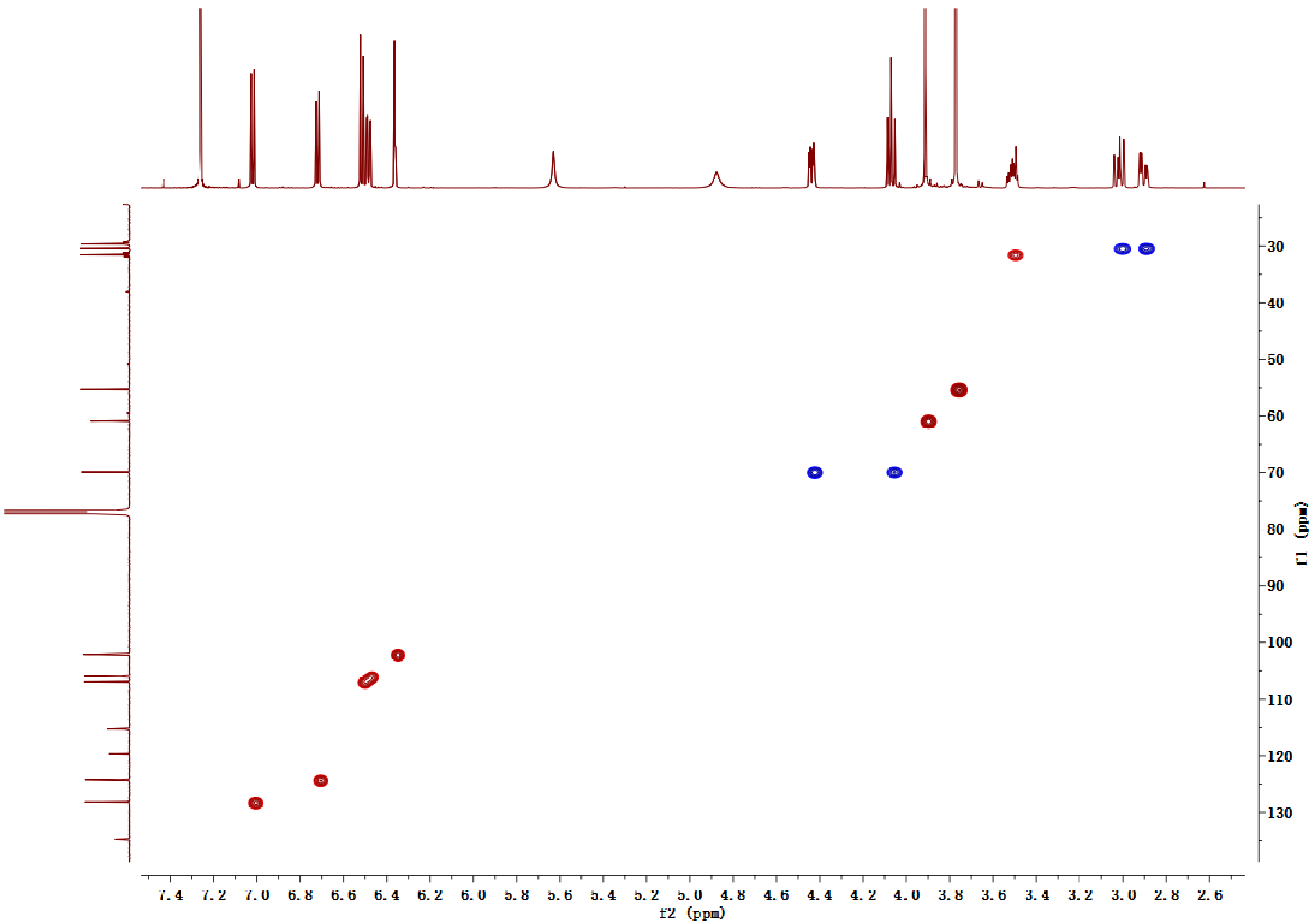

Figure S21. The HSQC Spectrum of Compound **3** in $CDCl_3$.

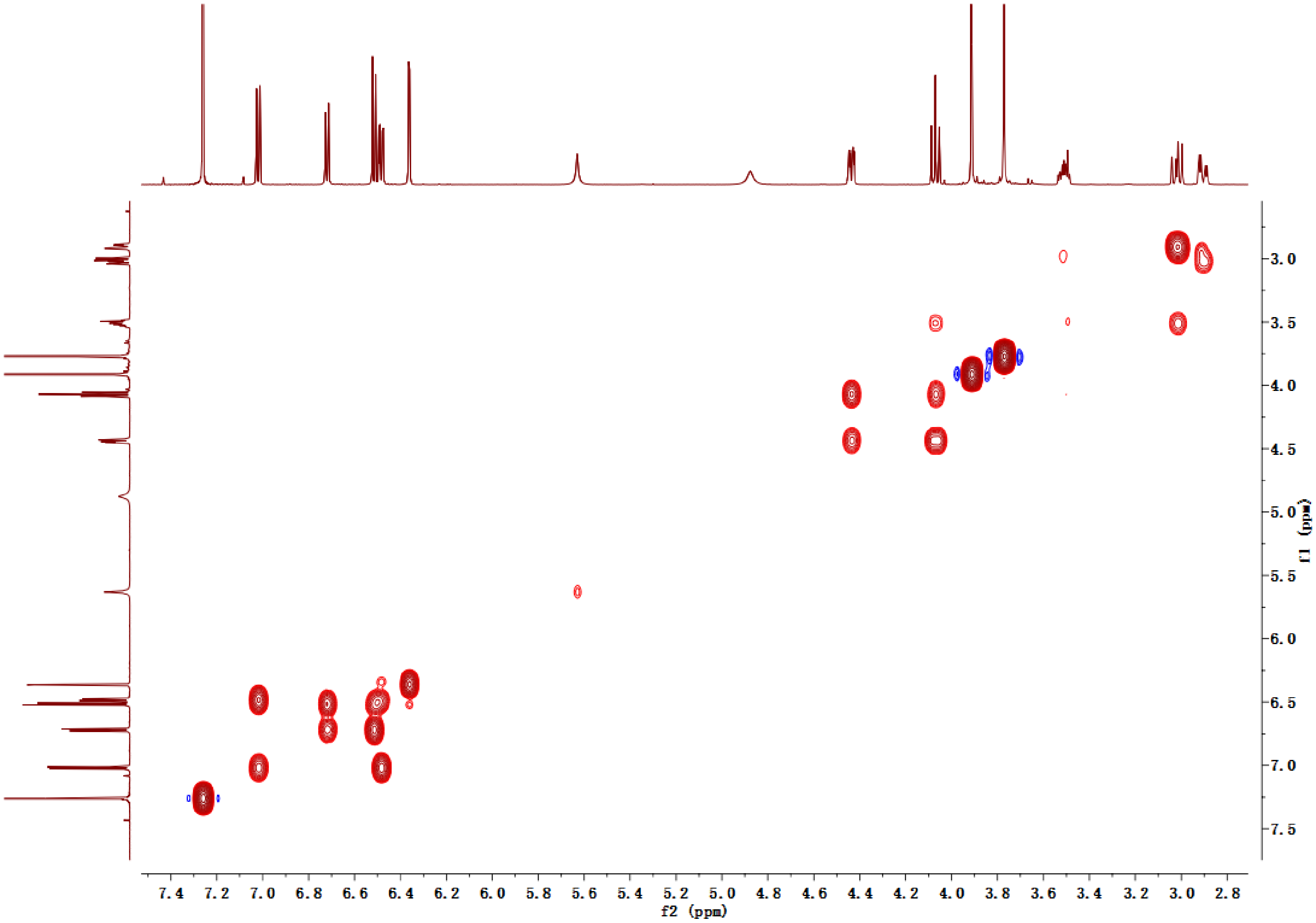

Figure S22. The ^{1}H-^{1}H gCOSY Spectrum of Compound **3** in $CDCl_3$.

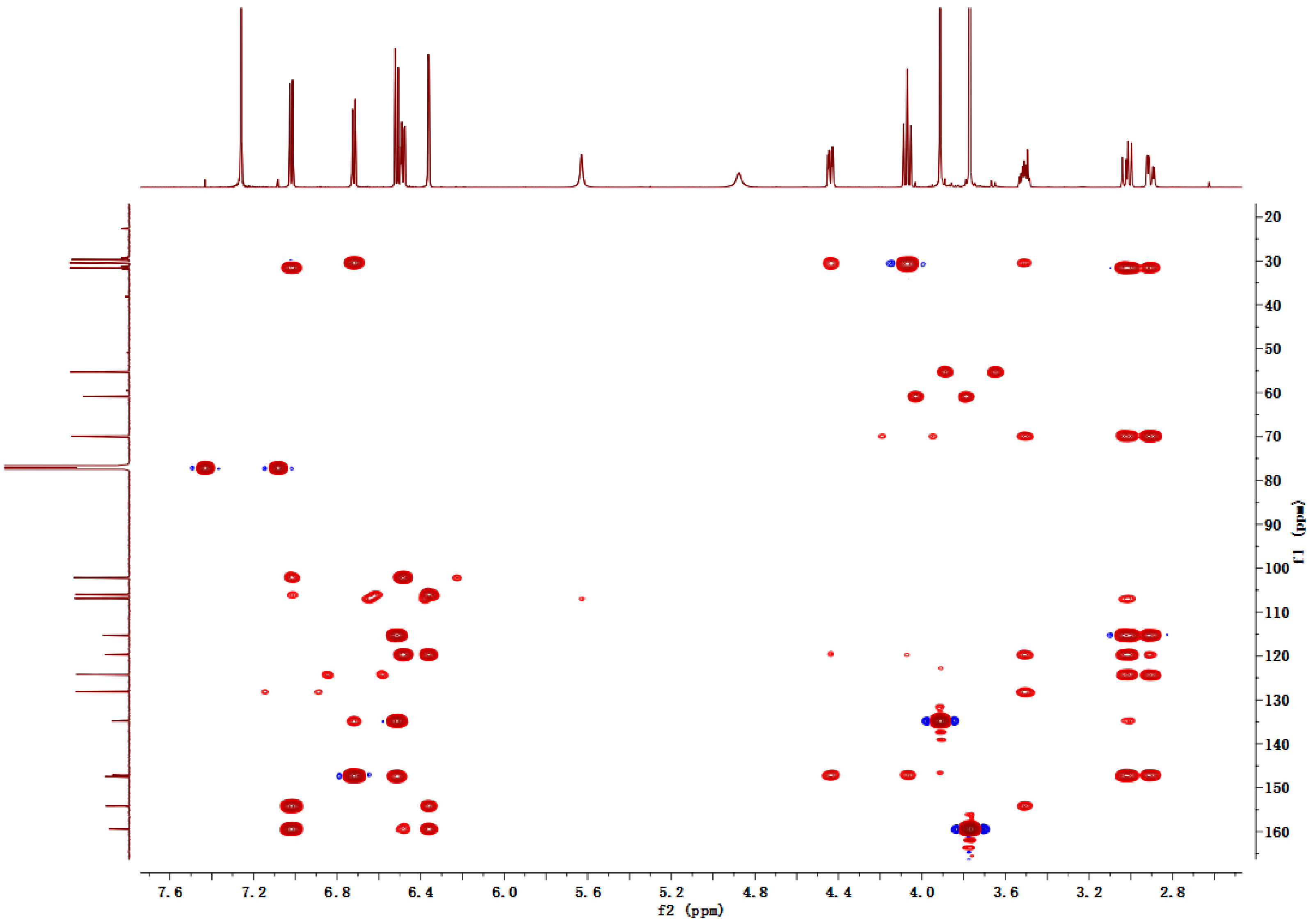

Figure S23. The HMBC Spectrum of Compound **3** in $CDCl_3$.

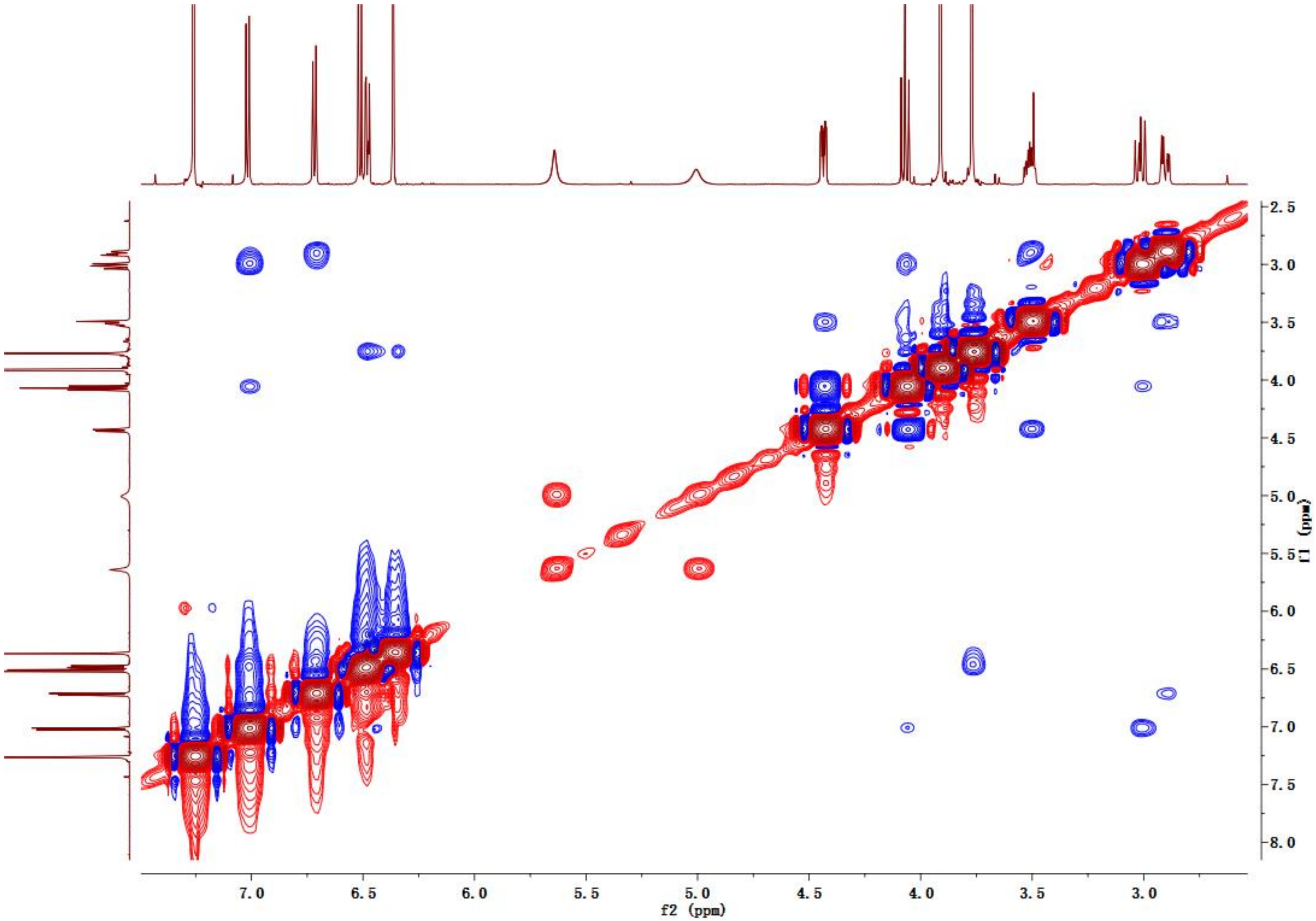

Figure S24. The NOESY Spectrum of Compound **3** in $CDCl_3$.

Single Mass Analysis
Tolerance = 3.0 mDa / DBE: min = -1.5, max = 50.0
Element prediction: Off
Number of isotope peaks used for i-FIT = 5
Monoisotopic Mass, Even Electron Ions
133 formula(e) evaluated with 2 results within limits (up to 50 closest results for each mass)
Elements Used:

Mass	Calc. Mass	mDa	PPM	DBE	Formula	i-FIT	i-FIT Norm	Fit Conf %	C	H	O	Na
351.0473	351.0481	-0.8	-2.3	11.5	C17 H12 O7 Na	561.4	0.288	74.97	17	12	7	1
	351.0446	2.7	7.7	23.5	C26 H7 O2	562.5	1.385	25.03	26	7	2	

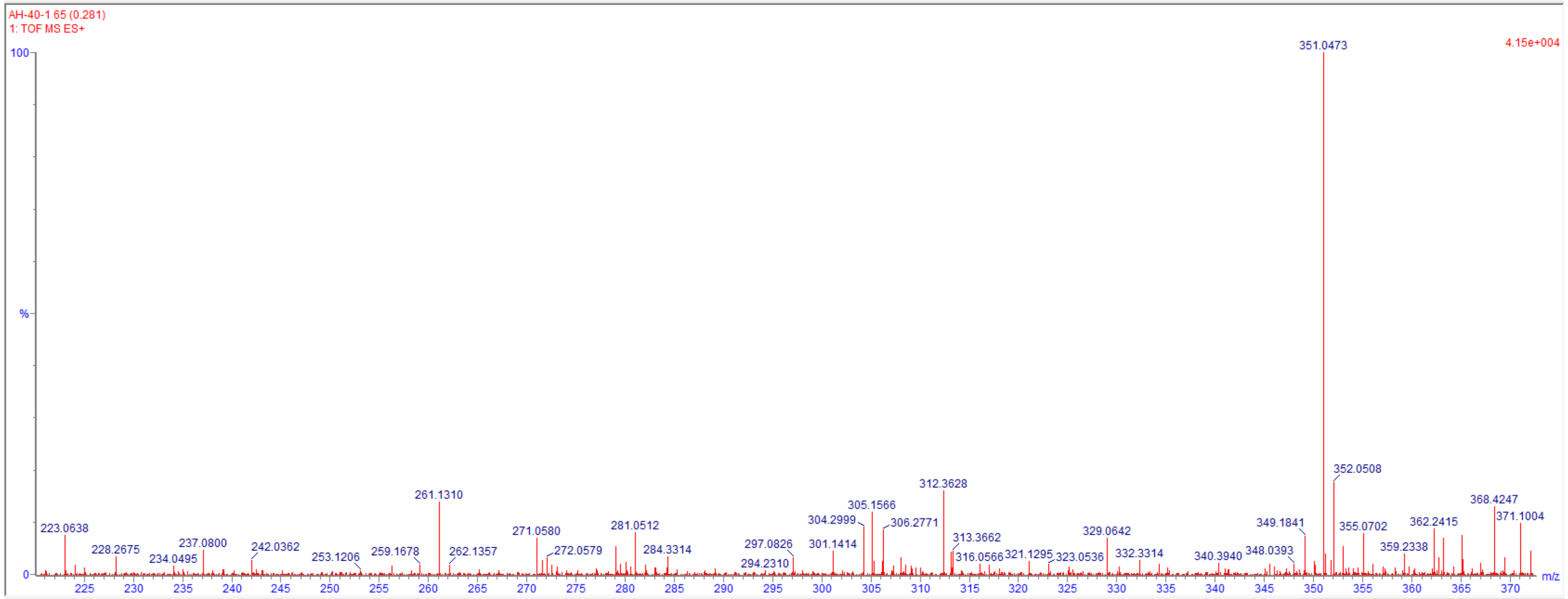

Figure S25. The (+)-HRESIMS Spectroscopic Data of Compound **5.**

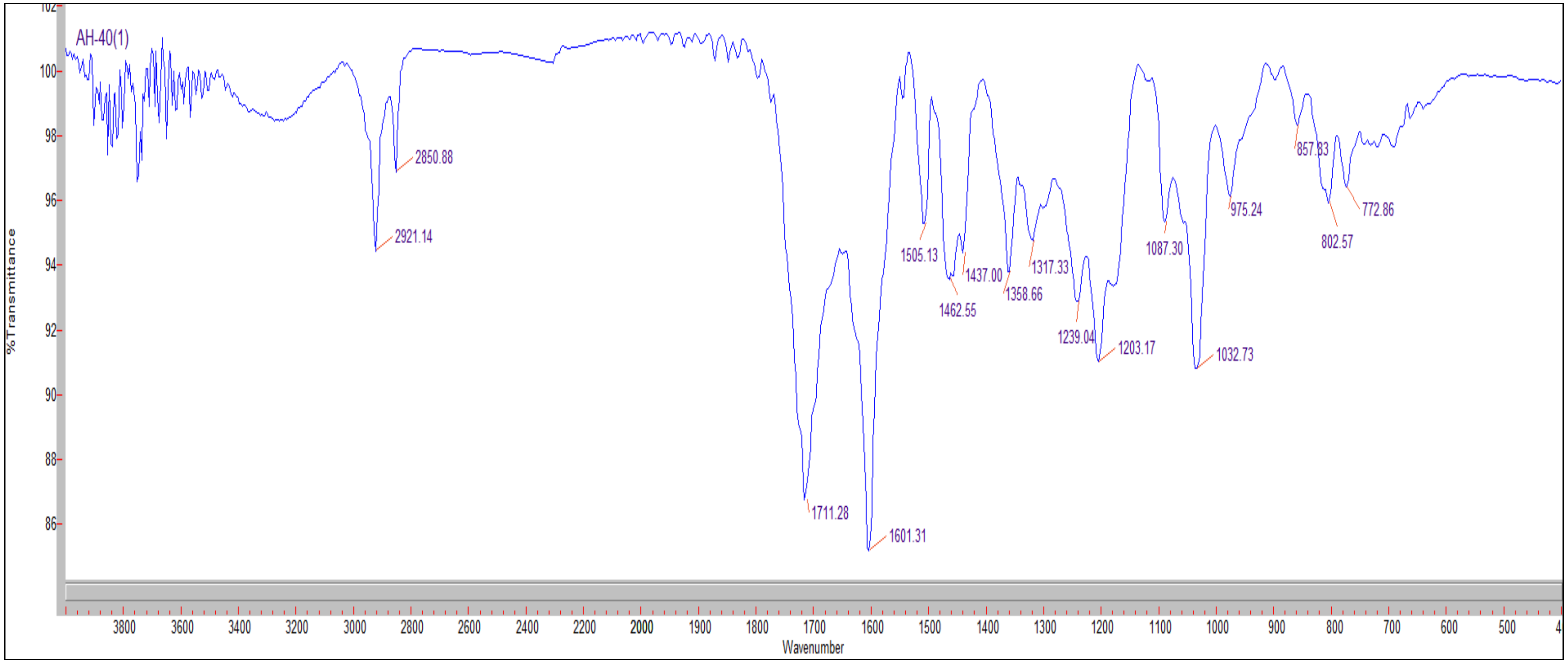

Figure S26. The IR Spectrum of Compound **5**.

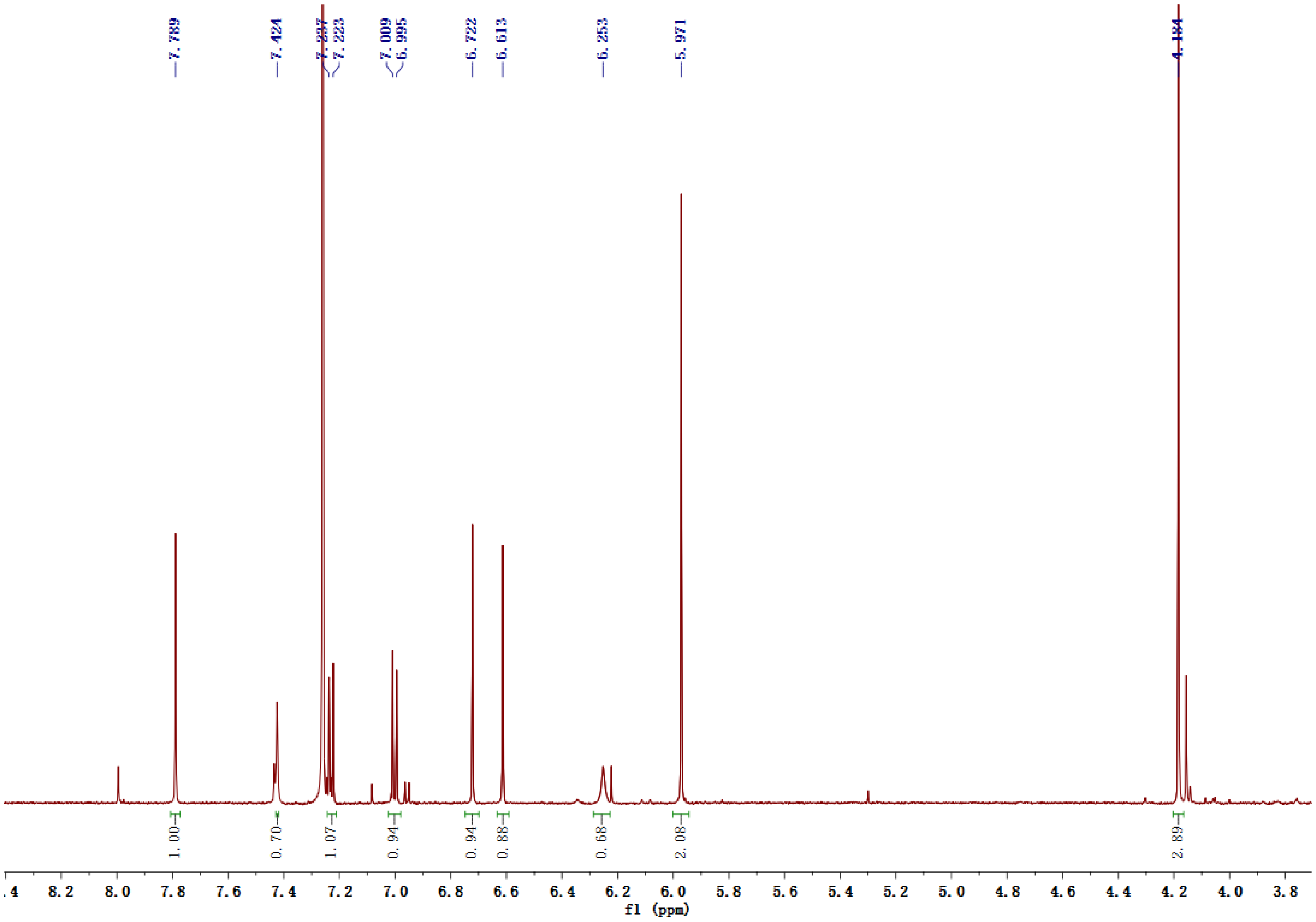

Figure S27. The ^{1}H NMR Spectrum of Compound **5** in $CDCl_3$.

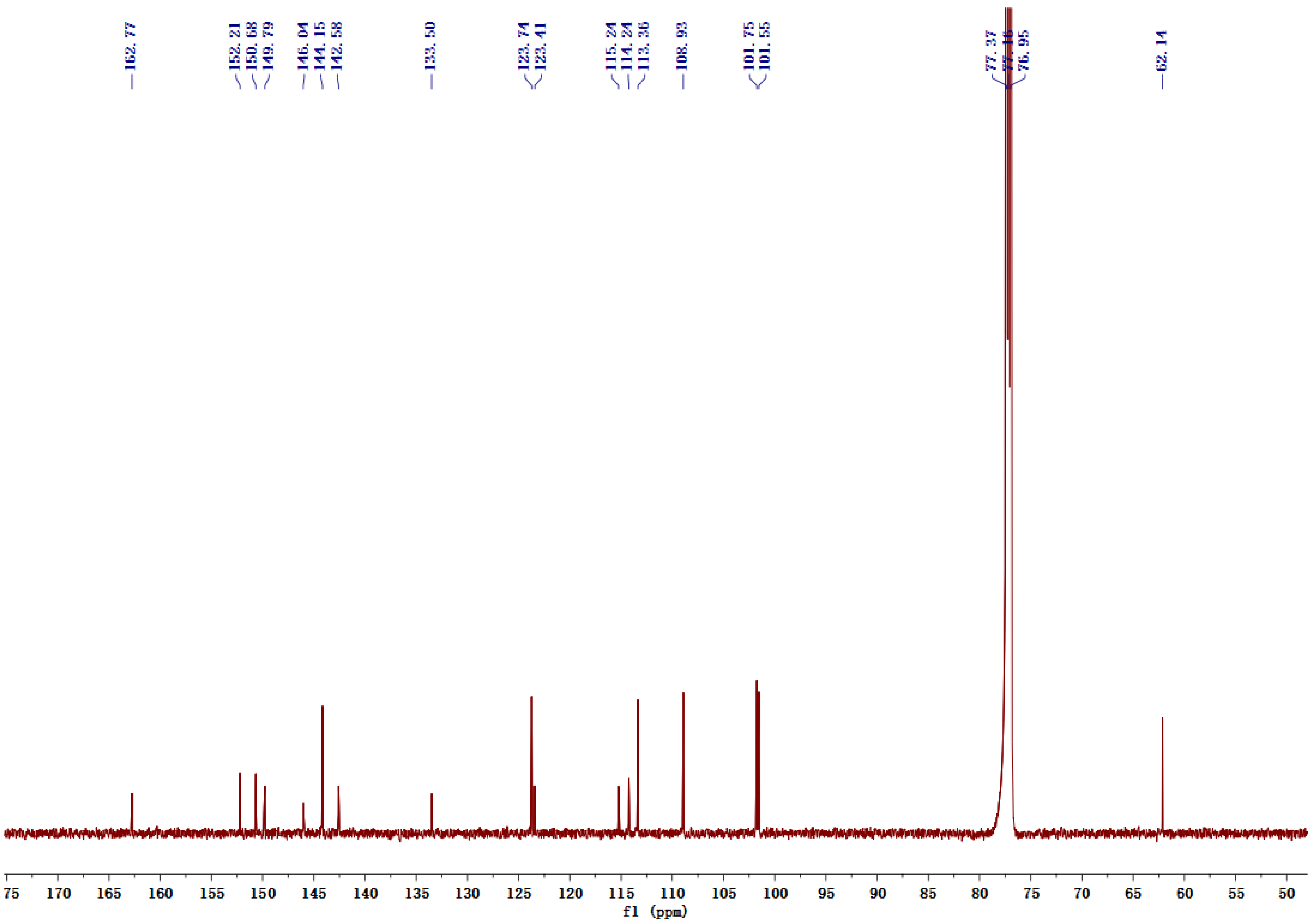

Figure S28. The ^{13}C NMR Spectrum of Compound **5** in $CDCl_3$.

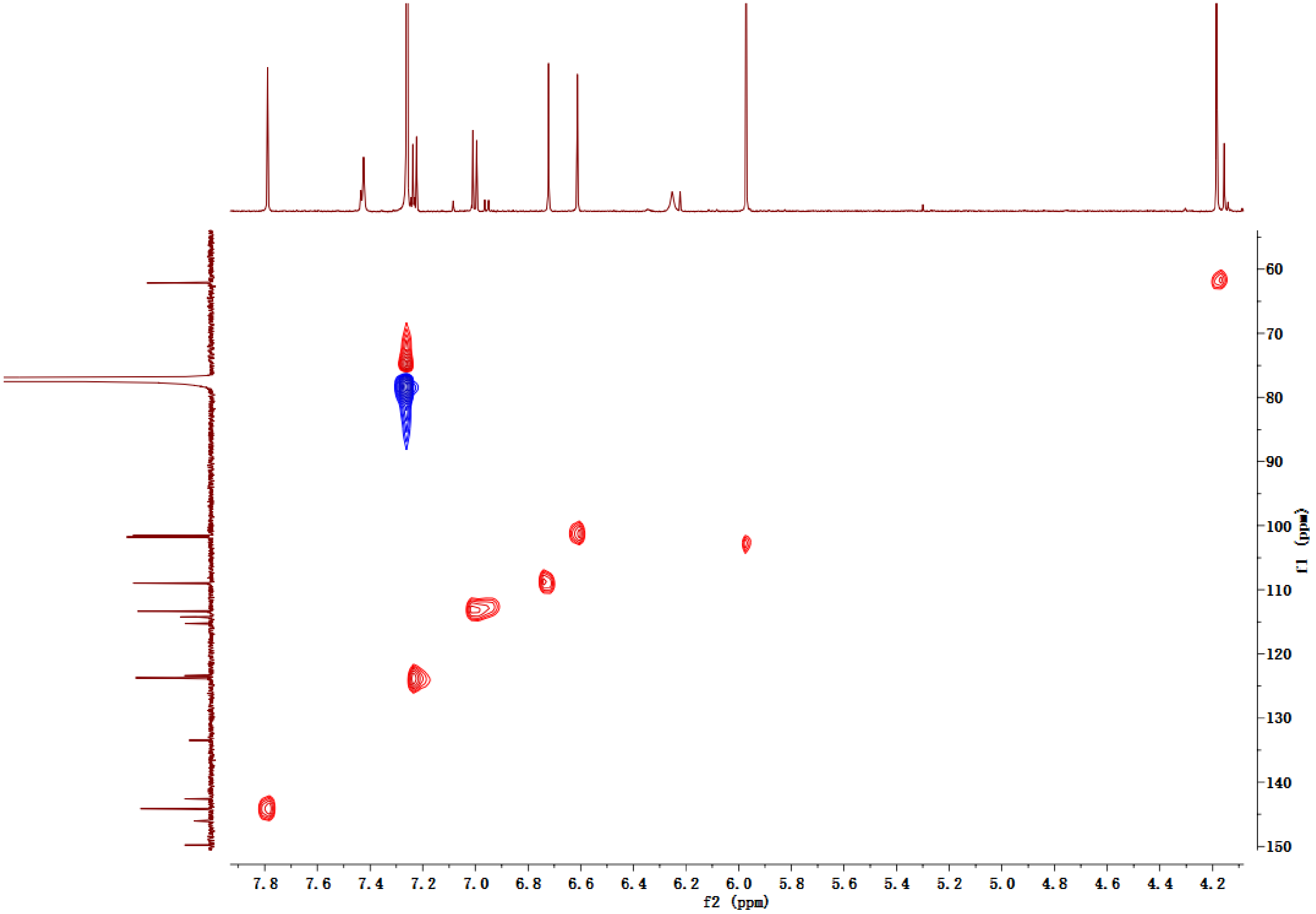

Figure S29. The HSQC Spectrum of Compound **5** in $CDCl_3$.

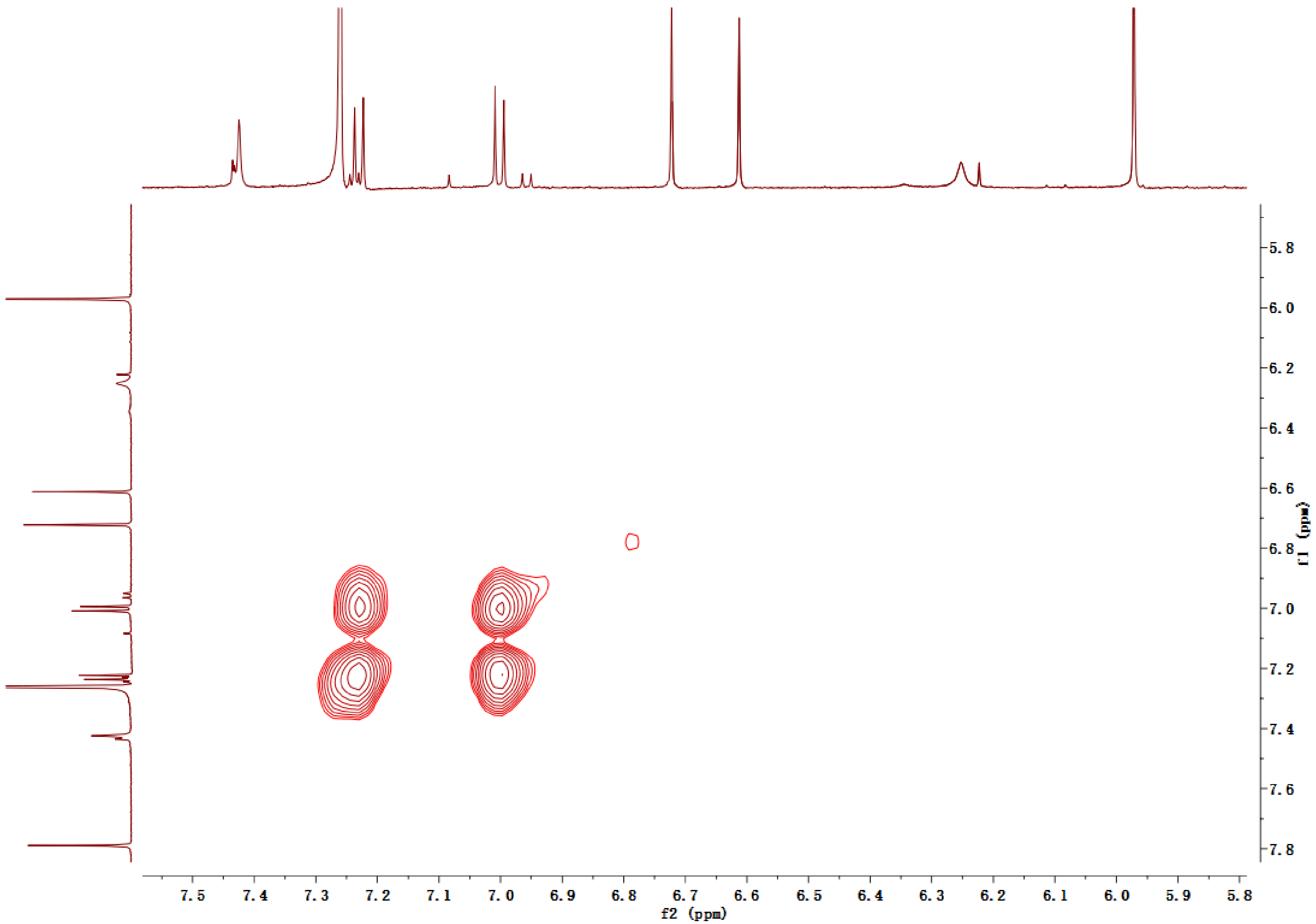

Figure S30. The ^{1}H-^{1}H gCOSY Spectrum of Compound **5** in $CDCl_3$.

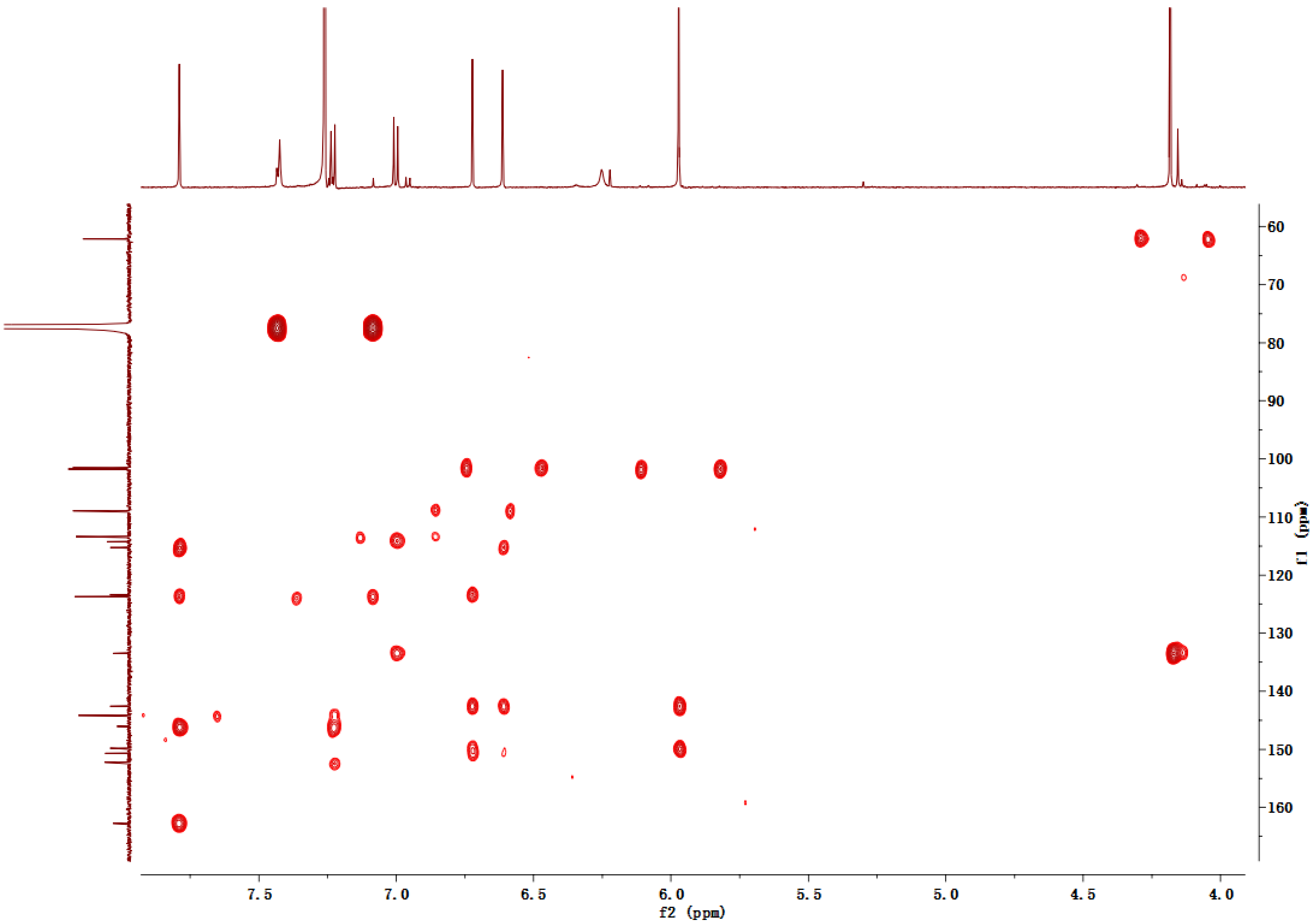

Figure S31. The HMBC Spectrum of Compound **5** in $CDCl_3$.
